# About Inverse Laplace Transform of a Dynamic Viscosity Function

**DOI:** 10.3390/ma15124364

**Published:** 2022-06-20

**Authors:** Kamil Urbanowicz, Anton Bergant, Rafał Grzejda, Michał Stosiak

**Affiliations:** 1 Faculty of Mechanical Engineering and Mechatronics, West Pomeranian University of Technology, 70-310 Szczecin, Poland; rafal.grzejda@zut.edu.pl; 2 Litostroj Power d.o.o., 1000 Ljubljana, Slovenia; anton.bergant@litostrojpower.eu; 3 Faculty of Mechanical Engineering, University of Ljubljana, 1000 Ljubljana, Slovenia; 4 Faculty of Mechanical Engineering, Wrocław University of Science and Technology, 50-370 Wrocław, Poland; michal.stosiak@pwr.edu.pl

**Keywords:** inverse Laplace transform, analytical solution, water hammer, dynamic viscosity function, fluid dynamics, pipe flow, Calogero–Ahmed sums

## Abstract

A dynamic viscosity function plays an important role in water hammer modeling. It is responsible for dispersion and decay of pressure and velocity histories. In this paper, a novel method for inverse Laplace transform of this complicated function being the square root of the ratio of Bessel functions of zero and second order is presented. The obtained time domain solutions are dependent on infinite exponential series and Calogero–Ahmed summation formulas. Both of these functions are based on zeros of Bessel functions. An analytical inverse will help in the near future to derive a complete analytical solution of this unsolved mathematical problem concerning the water hammer phenomenon. One can next present a simplified approximate form of this solution. It will allow us to correctly simulate water hammer events in large ranges of water hammer number, e.g., in oil–hydraulic systems. A complete analytical solution is essential to prevent pipeline failures while still designing the pipe network, as well as to monitor sensitive sections of hydraulic systems on a continuous basis (e.g., against possible overpressures, cavitation, and leaks that may occur). The presented solution has a high mathematical value because the inverse Laplace transforms of square roots from the ratios of other Bessel functions can be found in a similar way.

## 1. Introduction

**Figure 1:**
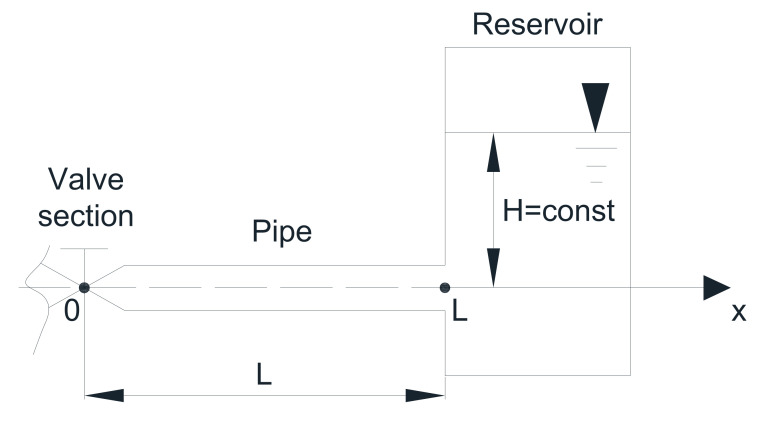
The reservoir–pipe–valve system.

Water hammer is propagation of pressure waves in liquid-filled pipelines due to the change of flow velocity. The phenomenon of water hammer has fascinated generations of researchers in academia and industry. The high interest is reflected in an increasing number of scientific works on this subject. In most works [1,2,3], numerical solutions are used when analyzing this phenomenon. However, commonly used numerical methods have only been verified experimentally over the years. Hence, an analytical method would be very useful. It would enable the theoretical verification of these solutions in the full range of variability of the basic flow parameters. A detailed review of the literature carried out in Reference [4] indicated that a complete laminar flow analytical solution, which would correctly model relatively short- and long-lasting water hammer events in the time domain, has still not been presented (derived). Interestingly, such a solution is not known only for complex systems, but also for the simplest hydraulic system, such as a pressure reservoir–pipe–valve system (Figure 1).

As it has been found from the extensive review of the literature carried out for the purposes of this work, there is relatively little work strictly related to the analytical modeling of the water hammer phenomenon. Most of the theoretical work concerns solutions that have been determined by solving only one of the water hammer equations (equation of motion). Among such solutions, there are solutions for accelerated [5,6,7,8,9], decelerated [10], reverse [11], pulsating [12,13], and other types of flows [14,15,16,17,18,19]. Relatively recently, new solutions have been developed in which a variable viscosity has been introduced during the duration of unsteady pipe flow [20,21], and with the help of newly derived methods, the previously known laminar models can be extended to turbulent flows [22,23].

The pioneering research studies that concerned attempts to solve the system of equations of continuity and motion describing the problems of water hammer were the works of Joukowsky [24] and Allievi [25]. These authors did not take into account the friction forces; hence, they managed to obtain very simple analytical solutions. The first to become interested in friction was Wood [26], who derived a number of Laplace solutions for basic practical cases. His work was significantly expanded by Rich [27], who derived analytical formulas in Laplace and time domain for water hammer cases analyzed by Wood, i.e., with the assumption of no friction and friction varying in a quasi-steady manner. The unsteady (frequency-dependent) nature of hydraulic resistance was noticed firstly by Iberall [28]. Unfortunately, despite numerous attempts to account for the frequency-dependent friction into account in the late 1950s [29,30] and at the beginning of the 1960s [31,32], no one, except for Holmboe [33,34] and Zielke [35,36], succeeded in presenting a solution in the time domain. Unfortunately, Holmboe’s solution was too simplified, considering only the first period of the water hammer event, and in addition, the author focused only on the cross-section at the valve. On the other hand, Zielke presented an inverse Laplace transform of the weighting function suitable for the numerical modeling of the wall shear stress, but he did not work on a complete analytical solution. In the 1970s, an interesting solution was presented by Karam [37,38]. This theoretical solution was limited to the step response at a downstream end in a semi-infinite fluid line, combined with a two-port representation of a finite line. The major feature introduced by Karam is two “filters” in a finite line model, representing a convolution of their arbitrary inputs with the unit impulse response at the equivalent location in a semi-infinite line. Fortunately, the promising Holmboe’s model has received a decent correction by Muto and Takahashi [39].

The initial verification of this model presented in recent papers [4,40,41] showed its enormous usefulness, especially in modeling water hammer occurring in water pipe flows (relatively low-viscosity water supply systems). Unfortunately, in typical hydraulic systems, i.e., wherever the working fluid is hydraulic oil (water hammer number 0.05 > *Wh* > 0.5 [42]), computational compliance was not sufficient. Further attempts of the analytical solution were undertaken by Sobey [43], whose solution, unfortunately, does not take into account the unsteady friction, and Mei-Jing [44,45]. The Mei-Jing model is the other promising model. It has been recently corrected in Reference [4] so that the asymptotic pressure values coincide with the expected final pressure value, even in the range of high values of the water hammer number. In the same conference paper, the authors showed other possible mathematical forms of this solution that turned out to be mathematically close to Rich’s quasi-steady model. The analysis of the Mei-Jing model showed its great potential, and, with a slight correction of the function describing the variability of the arguments, it seems that it will be (in the near future) the most accurate approximate model of the water hammer. It should be pointed out that recent research shows that the Atangana–Baleanu derivative and the Laguerre polynomial can be used to define a new computational technique for solving different types of differential equations [46]. The proposed scheme gives better convergence to the actual solution than the results available in the literature. It appears that use of the q-homotopy analysis algorithm in combination with the Laplace transform [47] can be a powerful tool to find new approximate solutions for this problem. In References [48,49,50,51], the authors proved that the finite element method is the most powerful scheme to compute the solutions of highly nonlinear ODEs.

Analytical solutions originally developed by Holmboe [33,34], which were later modified by Muto-Takahashi [39], are also the basis for the analysis of dynamic pressure and velocity waveforms, with the help of solutions commonly referred to as transmission line modelling. Interesting papers in this regard have been published by Oldenburger and Goodson [52], Brown [53], Viersma [54], and Ham [55]. These models are still very popular today, as exemplified by works of Hullhender [56], Johnston et al. [57], Krus [58], and Manhartsgruber [59]. The main role in these models plays the dynamic viscosity function. The inverse Laplace transform of it is the subject of this work. The presented method of the inverse Laplace transform of this physically important (in unsteady fluid pipe flow problems) dynamic viscosity function being the square root of the ratios of Bessel functions has not yet been presented in the literature (to the best knowledge of the authors). The novel method, after the expansion, will be helpful in finding similar inverse transforms from fractional ratios of Bessel functions.

Before we derive a new inverse Laplace solution, we briefly describe the problems of water hammer modeling. The necessary boundary conditions, which enabled Holmboe [33] to solve the problem in the Laplace domain, are here formulated. The frequency domain pressure solution is enhanced with the solution for the second basic parameter, i.e., the flow velocity. The complexity of a complete Laplace solution is the effect of nesting a square root of ratios of Bessel-based function in the arguments of hyperbolic functions. These nested functions are the main source accounting for the lack of a specific time domain analytical solution. The aim of this work is to show some new forms of the existing solutions from which it is possible to define a complete analytical solution in the Laplace domain. The presented solutions have not been discussed and published before.

Our next step is finding an inverse Laplace transform of the dynamic viscosity function. The analytical time domain form of this function has not been investigated before. This dynamic viscosity function plays the most important role in the presented final solutions because it is responsible for dispersion and decay of pressure and velocity histories. A novel double infinite formulation for the dynamic viscosity function is derived. Inverse Laplace transform is performed with the help of the convolutional integrals’ technique. In the new time domain solution, one can find a new type of unknown polynomial that is the result of the use of Calogero–Ahmed identities [60,61,62] for infinite series in the time domain, based on zeros of Bessel functions. In the near future, the novel solution will be used to find a complete analytical solution for any type of flow found in the analysis of unsteady pipe flows.

## 2. Water Hammer Equations

The equations that describe the water hammer phenomenon occurring in the simplest system presented in Figure 1 for the case of sudden valve closure are partial differential equations of the hyperbolic type. Holmboe [33], neglecting the dispersive term $\frac{\partial^{2}v}{\partial x^{2}}$, wrote the momentum equation in axial direction as follows:

(1) $$\frac{\partial \bar{v}}{\partial t}=-\frac{1}{\rho }\frac{\partial \bar{p}}{\partial x}+\frac{\nu }{r}\frac{\partial }{\partial r}\left(r\frac{\partial \bar{v}}{\partial r}\right),$$

where $\bar{v}=\bar{v}\left(x,r,t\right)$, and $\bar{p}=\bar{p}\left(x,t\right)$.

The averaged continuity equation was written by Holmboe as follows:

(2) $$\frac{\partial p}{\partial t}+\rho c^{2}\frac{\partial v}{\partial x}=0.$$

In the above continuity equation, the radial dependence was eliminated:

(3) $$\left[\begin{array}{c} v\\ p \end{array}\right]=\frac{1}{\pi R^{2}}\int_{0}^{R}2\pi r\left[\begin{array}{c} \bar{v}\\ \bar{p} \end{array}\right]dr,$$

and the mean quantities of pressure p and velocity v were defined.

A typical phenomenon of water hammer takes place when the fluid flow is suddenly blocked (e.g., by the sudden closing of the valve (see Figure 1) at the cross-section *x* = 0), and then intense pressure pulsations occur with values significantly exceeding the values recorded during the steady state flow. There is a potential hazard: the possibility of unsealing, leakage, and, in the worst case, rupture of the pipe wall. When the valve is closed, the kinetic energy of the flowing liquid stream changes into pressure energy. At the upstream end of the closing valve, a wave of an increased pressure is generated which travels along the length of the pipe at the pressure wave speed, *c*. This wave, after reaching the pressure reservoir, is reflected and travels back toward the valve. After returning to the valve, it reflects again as a negative pressure wave, and then it travels to the reservoir, where the second rebound takes place, and the wave returns back to the closed valve. After the second arrival to the valve, the first period of the water hammer event is completed, and the phenomenon begins to proceed as at the initial phase, but under slightly different conditions, because during the first impact, the front of this wave was dispersed as a result of skin friction between the liquid and the pipe wall. In mathematical analysis, we need to know what happens at the beginning, i.e., before analyzed transient event. In our problem steady flow exists, a laminar flow take place, which velocity profile has a parabolic shape, as described by Equation (6). A linear pressure drop occurs at the same time along the length of the pipe.

Axial boundary conditions for the initial and transient laminar flow situations are as follows [33]:

(a) For velocity

(4) $$\{\begin{array}{c} v\left(0,0\right)=v_{0}\\ v\left(0,t\right)=0 \end{array},$$

(b) For pressure

(5) $$\{\begin{array}{c} \;p\left(L,0\right)=p\left(L,t\right)=const\\ p\left(0,0\right)=p\left(L,0\right)-\frac{8\mu v_{0}}{R^{2}} \end{array}.$$

The initial condition of the velocity profile, at *t* = 0, may be given as follows:

(6) $$\bar{v}\left(x,r,0\right)=-2v_{0}\left[1-{\left(\frac{r}{R}\right)}^{2}\right].$$

The radial boundary conditions (in pre-transient, as well as in unsteady flow) are as follows:

(a) at $r=0;\;\frac{\partial v}{\partial r}=0$;
(b) at $r=R;\;\bar{v}\left(x,R,0\right)=\bar{v}\left(x,R,t\right)=0$ (no-slip condition).

## 3. Solution of Water Hammer Problem in Laplace Domain

Holmboe, after taking the Laplace transform of Equations (1) and (2), derived the pressure solution in the Laplace domain [33]:

(7) $$\tilde{p}\left(x,s\right)=\mathbb{Z}\frac{M}{s}\left[e^{-\frac{x}{c}M\cdot s}-e^{-\frac{\left(2L-x\right)}{c}M\cdot s}-e^{-\frac{\left(2L+x\right)}{c}M\cdot s}+e^{-\frac{\left(4L-x\right)}{c}M\cdot s}+e^{-\frac{\left(4L+x\right)}{c}M\cdot s}-\ldots \right]+\frac{8\mu v_{0}}{sR^{2}}x,$$

where $\mathbb{Z}=\rho cv_{0}$ is the Joukowsky pressure-rise formula.

The equation for velocity solution was derived recently [4], with the help of Equation (7), and it reads as follows:

(8) $$\tilde{v}\left(x,s\right)=-\frac{v_{0}}{s}\left[-e^{-\frac{x}{c}M\cdot s}-e^{-\frac{\left(2L-x\right)}{c}M\cdot s}+e^{-\frac{\left(2L+x\right)}{c}M\cdot s}+e^{-\frac{\left(4L-x\right)}{c}M\cdot s}-e^{-\frac{\left(4L+x\right)}{c}M\cdot s}-\ldots \right]-\frac{v_{0}}{s}.$$

In the above equations, the most important role is played by the dynamic viscosity function, M, which is a Laplace operator expressed in the following way [54,55]:

(9) $$M=\sqrt{-\frac{J_{0}\left(i\sqrt{\frac{s}{\nu }}R\right)}{J_{2}\left(i\sqrt{\frac{s}{\nu }}R\right)}}=\sqrt{\frac{I_{0}\left(\sqrt{s\frac{R^{2}}{\nu }}\right)}{I_{2}\left(\sqrt{s\frac{R^{2}}{\nu }}\right)}},$$

where $I_{0}$ and $I_{2}$ are modified Bessel functions of the zero and second order, respectively.

From Ham’s dissertation [55], as well as from Brereton and Jing’s paper [63], one can find the inverse Laplace transform of a function being an argument of this square root:

(10) $${ℒ}^{-1}\left\{\frac{I_{0}\left(\sqrt{\frac{s}{\nu }}R\right)}{I_{2}\left(\sqrt{\frac{s}{\nu }}R\right)}\right\}=\delta \left(t\right)+8\frac{\nu }{R^{2}}+4\frac{\nu }{R^{2}}\sum_{i=1}^{\infty }e^{-\eta_{i}{}^{2}\frac{\nu }{R^{2}}t},$$

where η_i_ represents consecutive zeros of the first-kind Bessel functions of the second order, $J_{2}\left(\eta_{i}\right)=0$.

It is worth noting that the exponential infinite series contained in Equation (10) plays an important role in the numerical modeling of water hammer and, more precisely, in modeling the skin friction occurring between the flowing liquid and the walls of the conduit. This series was originally derived by Zielke [35,36] for the purposes of numerical calculations of the wall shear stress. To increase the convergence of this solution (limiting the number of terms), it can be written in the following exponential form [64]:

(11) $$\sum_{i=1}^{\infty }e^{-\eta_{i}{}^{2}\frac{\nu }{R^{2}}t}\approx \sum_{i=1}^{k}m_{i}e^{-n_{i}\hat{t}},$$

where: $\hat{t}=t\frac{\nu }{R^{2}}$ is a dimensionless time.

In the literature on the modeling of unsteady pipe flows, it has been adopted as the weighting function. With the time and appearance of the effective solutions of convolutional integrals, the approximate solutions of this function have been shown by a number of authors in their works on numerical calculations of water hammer [64,65,66,67,68]. The use of the weighting function in the form of a finite sum presented by the formula in Equation (11) increases the efficiency of numerical calculations.

If the Laplace transform of the function described by the formula in Equation (10) is performed, and the following equivalent form of the sub-elemental function is obtained:

(12) $$\frac{I_{0}\left(\sqrt{\frac{s}{\nu }}R\right)}{I_{2}\left(\sqrt{\frac{s}{\nu }}R\right)}=1+\frac{8}{s}\frac{\nu }{R^{2}}+4\frac{\nu }{R^{2}}\sum_{i=1}^{\infty }\frac{1}{\eta_{i}{}^{2}\frac{\nu }{R^{2}}+s}.$$

The first two terms on the right-hand side of Equation (12) are quasi-steady solutions to the problem of fluid flow in a pressurized conduit (a linear friction model) that represent uniformly distributed resistance [58,69]. Thus one can write down the following:

(13) $$M_{q-s}=\sqrt{1+\frac{8}{s}\frac{\nu }{R^{2}}}.$$

**Figure 2:**
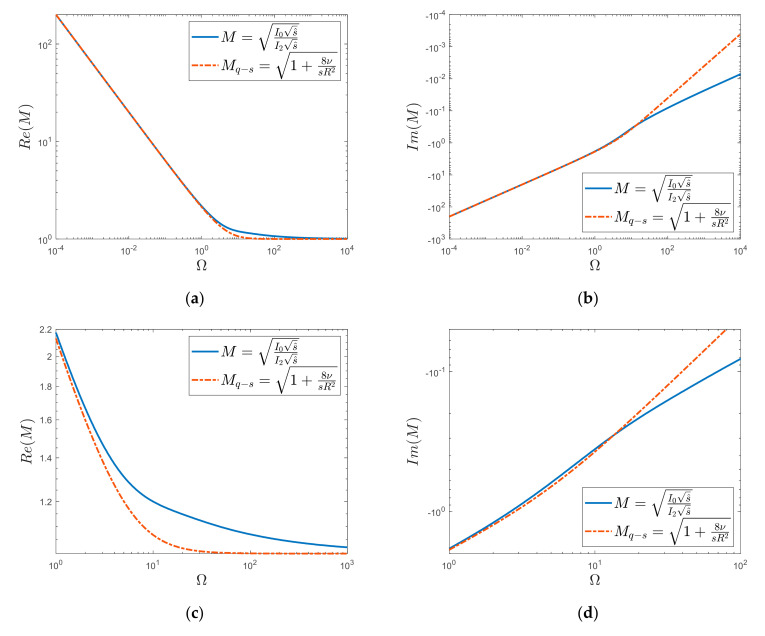
Course of dynamic viscosity function (**a**) Realis graph, (**b**) Imaginaris graph, (**c**) Enlarge of Realis graph, (**d**) Enlarge of Imaginaris graph.

Let us graphically compare the courses of both complete solutions (Equations (9) and (13)) by taking into account the frequency-dependent friction and the quasi-steady one. For the purpose of the graphical representation of the obtained results, let us define the dimensionless Laplace transform operator as $\hat{s}=s\frac{R^{2}}{\nu }$. Then, by replacing the Laplace transform with the Fourier transform, i.e., substituting for $\hat{s}=j\Omega$, (where Ω represents the dimensionless frequency $\Omega =\omega \frac{R^{2}}{\nu }$), it is possible to graphically analyze the above solutions (Figure 2). From Figure 2, it can be seen that the real part of the dynamic viscosity function, *M*, takes infinite values when $\hat{s}\to 0$; otherwise, i.e., when $\hat{s}\to \infty$, it goes to 1. The imaginary part goes from minus infinity to the zero value when $\hat{s}\to \infty$. The quasi-steady solution behaves similarly asymptotically, although there are significant differences between the waveforms of the *M* and *M_q-s_* functions, especially in the dimensionless frequency range from about 1 to 1000. The Realis of the quasi-steady solution, as can be seen clearly from Figure 2c, takes the final value (≈1) already for Ω≈30, while the unsteady solution has values greater than 1, still even for the frequency Ω≈1000. The imaginary waveforms (Figure 2d) confirm what is visible in the real-part waveforms, i.e., that both of these functions at different times tend to asymptotic (end) values. Hence, in Figure 2d, from the frequency Ω≈10, it can be seen that these functions tend to the final values with a different dynamic.

## 4. Novel Method to Calculate the Inverse Laplace Transform of *M* Function

Let us use the following integral representation (special thanks for pointing this identity goes to Professor Alireza Ansari from Shahrekord University, Iran):

(14) $$\frac{1}{\sqrt{s^{*}}}=\frac{1}{\sqrt{\pi }}\int_{0}^{\infty }\frac{e^{-s^{*}\tau }}{\sqrt{\tau }}d\tau ,$$

where:

(15) $$s^{*}=\frac{I_{2}\left(\sqrt{s\frac{R^{2}}{\nu }}\right)}{I_{0}\left(\sqrt{s\frac{R^{2}}{\nu }}\right)}=-\frac{J_{2}\left(i\sqrt{\frac{s}{\nu }}R\right)}{J_{0}\left(i\sqrt{\frac{s}{\nu }}R\right)}.$$

With the help of Brereton–Jiang [63] solution, we achieve the following:

(16) $$\frac{I_{2}\left(\sqrt{s\frac{R^{2}}{\nu }}\right)}{I_{0}\left(\sqrt{s\frac{R^{2}}{\nu }}\right)}=s^{2}\frac{R^{2}}{\nu }\left(\frac{1}{8s}-4\sum_{i=1}^{\infty }\frac{1}{\mu_{i}^{6}\frac{\nu }{R^{2}}+\mu_{i}^{4}s}\right),$$

where $\mu_{i}$ represents the consecutive zeros of the first-kind Bessel function of the zero order, $J_{0}\left(\mu_{i}\;\right)=0$.

Let use a partial fraction expansion to the right-hand side of the above equation; then one obtains the following form:

(17) $$s^{2}\frac{R^{2}}{\nu }\left(\frac{1}{8s}-4\sum_{i=1}^{\infty }\frac{1}{\mu_{i}^{6}\frac{\nu }{R^{2}}+\mu_{i}^{4}s}\right)=1-4\sum_{i=1}^{\infty }\frac{1}{\mu_{i}^{2}+\frac{R^{2}}{\nu }s}.$$

Through the combination and rearrangement of Equations (14)–(17), we obtain the following:

(18) $$\frac{1}{\sqrt{\frac{I_{2}\left(\sqrt{s\frac{R^{2}}{\nu }}\right)}{I_{0}\left(\sqrt{s\frac{R^{2}}{\nu }}\right)}}}=\frac{1}{\sqrt{\pi }}\int_{0}^{\infty }\frac{e^{-s^{2}\frac{R^{2}}{\nu }\left(\frac{1}{8s}-4\sum_{i=1}^{\infty }\frac{1}{\mu_{i}^{6}\frac{\nu }{R^{2}}+\eta_{i}^{4}s}\right)\tau }}{\sqrt{\tau }}d\tau =\frac{1}{\sqrt{\pi }}\int_{0}^{\infty }\frac{e^{-\left(1-4\sum_{i=1}^{\infty }\frac{1}{\mu_{i}^{2}+\frac{R^{2}}{\nu }s}\right)\tau }}{\sqrt{\tau }}d\tau ,$$

and by working on the last term of the right-hand side of Equation (18), we achieve the following:

(19) $$\int_{0}^{\infty }\frac{e^{-\left(1-4\sum_{i=1}^{\infty }\frac{1}{\mu_{i}^{2}+\frac{R^{2}}{\nu }s}\right)\tau }}{\sqrt{\tau }}d\tau =\int_{0}^{\infty }\frac{e^{-\tau }e^{4\tau \sum_{i=1}^{\infty }\frac{1}{\mu_{i}^{2}+\frac{R^{2}}{\nu }s}}}{\sqrt{\tau }}d\tau =\int_{0}^{\infty }\frac{e^{-\tau }\sum_{k=0}^{\infty }\frac{{\left(4\tau \frac{\nu }{R^{2}}\sum_{i=1}^{\infty }\frac{1}{\mu_{i}^{2}\frac{\nu }{R^{2}}+s}\right)}^{k}}{k!}}{\sqrt{\tau }}d\tau =\;\sum_{k=0}^{\infty }\frac{4^{k}}{k!}\int_{0}^{\infty }\frac{e^{-\tau }\tau^{k}{\left(\frac{\nu }{R^{2}}\right)}^{k}{\left(\sum_{i=1}^{\infty }\frac{1}{\mu_{i}^{2}\frac{\nu }{R^{2}}+s}\right)}^{k}}{\sqrt{\tau }}d\tau =\sum_{k=0}^{\infty }\frac{4^{k}}{k!}\int_{0}^{\infty }e^{-\tau }\tau^{k-0.5}{\left(\frac{\nu }{R^{2}}\right)}^{k}{\left(\sum_{i=1}^{\infty }\frac{1}{\mu_{i}^{2}\frac{\nu }{R^{2}}+s}\right)}^{k}d\tau =\;\sum_{k=0}^{\infty }\frac{4^{k}}{k!}{\left(\frac{\nu }{R^{2}}\right)}^{k}{\left(\sum_{i=1}^{\infty }\frac{1}{\mu_{i}^{2}\frac{\nu }{R^{2}}+s}\right)}^{k}\int_{0}^{\infty }e^{-\tau }\tau^{k-0.5}d\tau =\sum_{k=0}^{\infty }\frac{4^{k}}{k!}{\left(\frac{\nu }{R^{2}}\right)}^{k}{\left(\sum_{i=1}^{\infty }\frac{1}{\mu_{i}^{2}\frac{\nu }{R^{2}}+s}\right)}^{k}\left(\Gamma \left(k+0.5,0\right)-\Gamma \left(k+0.5,\infty \right)\right)$$

where Γ(b,t) is the incomplete gamma function which for t=∞ has a zero value, Γ(b,∞)=0, and one finally obtains the following:

(20) $$\sqrt{\frac{I_{0}\left(\sqrt{s\frac{R^{2}}{\nu }}\right)}{I_{2}\left(\sqrt{s\frac{R^{2}}{\nu }}\right)}}=\frac{1}{\sqrt{\pi }}\sum_{k=0}^{\infty }\frac{\Gamma \left(k+0.5\right)}{\Gamma \left(k+1\right)}{\left(\frac{4\nu }{R^{2}}\right)}^{k}{\left(\sum_{i=1}^{\infty }\frac{1}{\mu_{i}^{2}\frac{\nu }{R^{2}}+s}\right)}^{k}.$$

The above form based on the gamma function (Γ(k+0.5) and Γ(k+1)) of the solution is useful in our further derivations in this paper. The above Equation (20) can also be written in two other forms, respectively, dependent on binomial function and factorials:

(21) $$\sqrt{\frac{I_{0}\left(\sqrt{s\frac{R^{2}}{\nu }}\right)}{I_{2}\left(\sqrt{s\frac{R^{2}}{\nu }}\right)}}=\sum_{k=0}^{\infty }\left(\begin{array}{c} 2k\\ k \end{array}\right){\left(\frac{\nu }{R^{2}}\sum_{i=1}^{\infty }\frac{1}{\mu_{i}^{2}\frac{\nu }{R^{2}}+s}\right)}^{k}=\sum_{k=0}^{\infty }\frac{\left(2k\right)!}{k{!}^{2}}{\left(\frac{\nu }{R^{2}}\sum_{i=1}^{\infty }\frac{1}{\mu_{i}^{2}\frac{\nu }{R^{2}}+s}\right)}^{k},$$

where $\left(\begin{array}{c} n\\ k \end{array}\right)$ is a binomial coefficient, which is defined as follows:

(22) $$\left(\begin{array}{c} n\\ k \end{array}\right)=\frac{n!}{\left(n-k\right)!k!}.$$

We do not bother with the quotient of the gamma function, the square root of π, or the term ${\left(\frac{4\nu }{R^{2}}\right)}^{k}$ presented in Equation (20) because they constitute a certain constant value depending on *k*. Thus, the problem of Laplace’s inverse transformation is based on finding inverses from the following power function:

(23) $${ℒ}^{-1}\left\{{\left(\sum_{i=1}^{\infty }\frac{1}{\mu_{i}^{2}\frac{\nu }{R^{2}}+s}\right)}^{k}\right\}.$$

The solution for *k* = 0 is δ(t) (Dirac delta function), while for *k* = 1, it is commonly known to be the following:

(24) $${ℒ}^{-1}\left\{\sum_{i=1}^{\infty }\frac{1}{\mu_{i}^{2}\frac{\nu }{R^{2}}+s}\right\}=\sum_{i=1}^{\infty }e^{-\mu_{i}^{2}\frac{\nu }{R^{2}}t}.$$

For *k* > 1, the following approach based on convolutional integrals is proposed:

(25) $$L^{-1}\left\{{\left(\sum_{i=1}^{\infty }\frac{1}{\mu_{i}^{2}\frac{\nu }{R^{2}}+s}\right)}^{k}\right\}=\left\{\begin{array}{c} A=\int_{0}^{t}\overset{\infty }{\underset{i=1}{\sum }}e^{-\mu_{j}^{2}\frac{\nu }{R^{2}}u}\overset{\infty }{\underset{j=1}{\sum }}e^{-\mu_{j}^{2}\frac{\nu }{R^{2}}u}du\;\;\;\;\;for\;\;k=2\\ B=\int_{0}^{t}A\left(t-u\right)\overset{\infty }{\underset{j=1}{\sum }}e^{-\mu_{j}^{2}\frac{\nu }{R^{2}}u}du\;\;\;\;\;for\;\;k=3\\ C=\int_{0}^{t}B\left(t-u\right)\overset{\infty }{\underset{j=1}{\sum }}e^{-\mu_{j}^{2}\frac{\nu }{R^{2}}u}du\;\;\;\;\;for\;\;k=4\\ D=\int_{0}^{t}C\left(t-u\right)\overset{\infty }{\underset{j=1}{\sum }}e^{-\mu_{j}^{2}\frac{\nu }{R^{2}}u}du\;\;\;\;\;for\;\;k=5 \end{array}\right..$$

To better understand what type of function we are dealing with after calculating the convolution, let us consider in detail a fairly simple case for *k* = 2. Let us simplify the problem by analyzing possible analytical solutions of this type of convolution:

(26) $$A^{*}=\int_{0}^{t}e^{-a\left(t-u\right)}e^{-bu}du,$$

where $a=\mu_{a}^{2}\frac{\nu }{R^{2}}$, and $b=\mu_{b}^{2}\frac{\nu }{R^{2}}$.

The result of the above integral depends on the values of the coefficients *a* and *b* (always positive in our case). A simpler solution is obtained when a=b, as then we obtain the following:

(27) $$A_{1}^{*}=\int_{0}^{t}e^{-a\left(t-u\right)}e^{-au}du=te^{-at}.$$

However, when a≠b, one achieves the following:

(28) $$A_{2}^{*}=\int_{0}^{t}e^{-a\left(t-u\right)}e^{-bu}du=\frac{e^{-bt}-e^{-at}}{a-b}=\frac{e^{-bt}}{a-b}-\frac{e^{-at}}{a-b}.$$

If, instead of a single product of exponential functions, we consider the product of infinite sums built on them, we get the following result for *k* = 2:

(29) $$A=t\sum_{i=1}^{\infty }e^{-\mu_{i}^{2}\frac{\nu }{R^{2}}t}+\frac{R^{2}}{\nu }\sum_{i=1}^{\infty }e^{-\mu_{i}^{2}\frac{\nu }{R^{2}}t}\left[\sum_{\begin{array}{c} j=1\\ \;j\neq i \end{array}}^{\infty }\frac{1}{\mu_{j}^{2}-\mu_{i}^{2}}-\sum_{\begin{array}{c} j=1\\ \;j\neq i \end{array}}^{\infty }\frac{1}{\mu_{i}^{2}-\mu_{j}^{2}}\right].$$

The sum of two infinite series based on the differences of zeros of the Bessel function, i.e., the square bracket, can be written as follows:

(30) $$\sum_{\begin{array}{c} j=1\\ \;j\neq i \end{array}}^{\infty }\frac{1}{\mu_{j}^{2}-\mu_{i}^{2}}-\sum_{\begin{array}{c} j=1\\ \;j\neq i \end{array}}^{\infty }\frac{1}{\mu_{i}^{2}-\mu_{j}^{2}}=\sum_{\begin{array}{c} j=1\\ \;j\neq i \end{array}}^{\infty }\frac{1}{\mu_{j}^{2}-\mu_{i}^{2}}+\sum_{\begin{array}{c} j=1\\ \;j\neq i \end{array}}^{\infty }\frac{1}{\mu_{j}^{2}-\mu_{i}^{2}}=2\sum_{\begin{array}{c} j=1\\ \;j\neq i \end{array}}^{\infty }\frac{1}{\mu_{j}^{2}-\mu_{i}^{2}}.$$

The infinite series of the above type was dealt with by Calogero [60,61] and then by Ahmed and Calogero [62] (the first author of this paper would like to thank Dr. Javier Garcia from the University of A Coruña, Spain, for pointing out this important relationship). In the first paper [60], Calogero proved that the infinite series based on Bessel functions satisfy the infinite set of equations:

(31) $$\sum_{\begin{array}{c} j=1\\ \;j\neq i \end{array}}^{\infty }\frac{1}{\mu_{j,h}^{2}-\mu_{i,h}^{2}}=\frac{1+h}{2\mu_{i,h}^{2}},$$

where *h* is the order of Bessel functions. The obtained results are related to the established connection between the motion of poles and zeros of special solutions of partial differential equations and solvable many-body problems. In the next paper [61], Calogero found that this infinite system (31), together with the Rayleigh sum rule, i.e.,

(32) $$\sum_{\begin{array}{c} i=1 \end{array}}^{\infty }\frac{1}{\mu_{i,h}^{2}}=\frac{1}{4\left(h+1\right)},$$

eliminates the scale invariance of (31) and characterizes the (squared) zeros of the Bessel function $J_{h}$, namely, that the solution of (31) and (32) is unique. The author next proved that other relations of a similar type can be derived:

(33) $$\sum_{\begin{array}{c} j=1\\ \;j\neq i \end{array}}^{\infty }\frac{1}{{\left(\mu_{j,h}^{2}-\mu_{i,h}^{2}\right)}^{2}}=\frac{\mu_{i,h}^{2}-\left(h+1\right)\left(h+5\right)}{12\mu_{i,h}^{4}},$$

(34) $$\sum_{\begin{array}{c} j=1\\ \;j\neq i \end{array}}^{\infty }\frac{1}{{\left(\mu_{j,h}^{2}-\mu_{i,h}^{2}\right)}^{3}}=-\frac{\mu_{i,h}^{2}-2\left(h+1\right)\left(h+3\right)}{16\mu_{i,h}^{6}}.$$

Other solutions of this, as well as of a modified type (denominators multiplied additionally by $\mu_{j,h}^{a}$), were presented in a coauthored paper by Ahmed and Calogero [62]. For other interesting relations of similar types, please consult References [70,71,72,73].

Using the Calogero solution, Equation (32), the above sum for the zeros of the Bessel function of the first kind and zero order can be written as follows (in our case *h* = 0):

(35) $$\sum_{\begin{array}{c} j=1\\ \;j\neq i \end{array}}^{\infty }\frac{1}{\mu_{j}^{2}-\mu_{i}^{2}}=\frac{1}{2\mu_{i}^{2}}.$$

The above identity significantly simplifies the obtained final solution for *k* = 2 in the time domain as follows:

(36) $$A=t\sum_{i=1}^{\infty }e^{-\mu_{i}^{2}\frac{\nu }{R^{2}}t}+\frac{R^{2}}{\nu }\sum_{i=1}^{\infty }\frac{e^{-\mu_{i}^{2}\frac{\nu }{R^{2}}t}}{\mu_{i}^{2}}.$$

The obtained result can be easily verified numerically by going back to the Laplace domain with this solution:

(37) $$ℒ\left\{A\right\}=\sum_{i=1}^{\infty }\frac{1}{{\left(\mu_{i}^{2}\frac{\nu }{R^{2}}+s\right)}^{2}}+\frac{R^{2}}{\nu }\sum_{i=1}^{\infty }\frac{1}{\mu_{i}^{2}\left(\mu_{i}^{2}\frac{\nu }{R^{2}}+s\right)}={\left(\sum_{i=1}^{\infty }\frac{1}{\mu_{i}^{2}\frac{\nu }{R^{2}}+s}\right)}^{2}.$$

The solution for *k* = 2 multiplied by ${\left(\frac{\nu }{R^{2}}\right)}^{k}$ has the following form:

(38) $${\left(\frac{\nu }{R^{2}}\sum_{i=1}^{\infty }\frac{1}{\mu_{i}^{2}\frac{\nu }{R^{2}}+s}\right)}^{2}={\left(\frac{\nu }{R^{2}}\right)}^{2}\sum_{i=1}^{\infty }\frac{1}{{\left(\mu_{i}^{2}\frac{\nu }{R^{2}}+s\right)}^{2}}+{\left(\frac{\nu }{R^{2}}\right)}^{2}\frac{R^{2}}{\nu }\sum_{i=1}^{\infty }\frac{1}{\mu_{i}^{2}\left(\mu_{i}^{2}\frac{\nu }{R^{2}}+s\right)}.$$

For the purposes of graphical comparative analysis, let us write it in a dimensionless form:

(39) $$\tilde{A}={\left(\sum_{i=1}^{\infty }\frac{1}{\mu_{i}^{2}+\hat{s}}\right)}^{2}=\sum_{i=1}^{\infty }\frac{1}{{\left(\mu_{i}^{2}+\hat{s}\right)}^{2}}+\sum_{i=1}^{\infty }\frac{1}{\mu_{i}^{2}\left(\mu_{i}^{2}+\hat{s}\right)},$$

**Figure 3:**
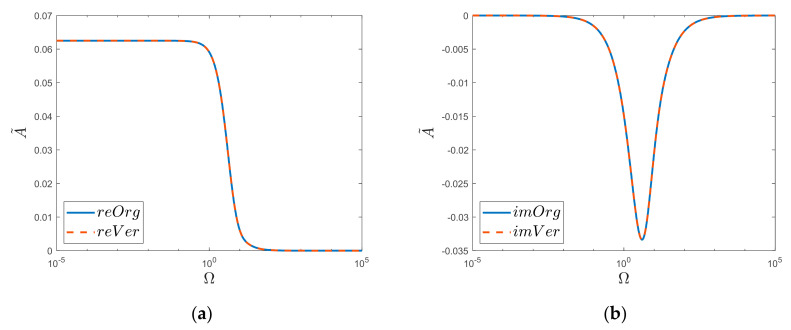
(**a**) Realis and (**b**) Imaginaris graphs of original function “*Org*” and newly derived substitute “*Ver*”. Semi−log scale for *k* = 2 case.

In this way, it is possible to graphically analyze the obtained solution in the frequency domain (Figure 3).

**Figure 4:**
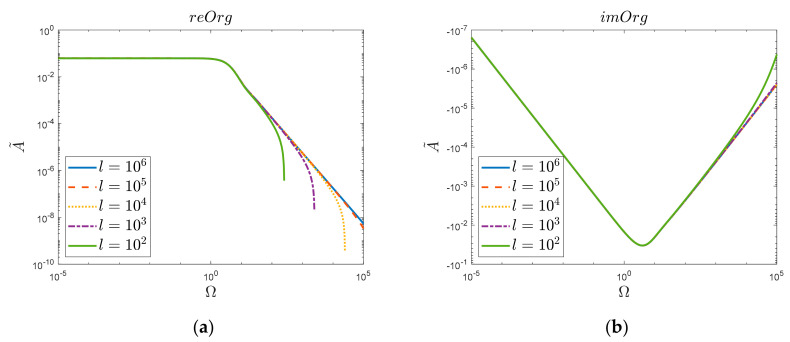
Dependence on the number of summation terms “*l*” of original function “*Org*”: (**a**) Realis, (**b**) Imaginaris.

**Figure 5:**
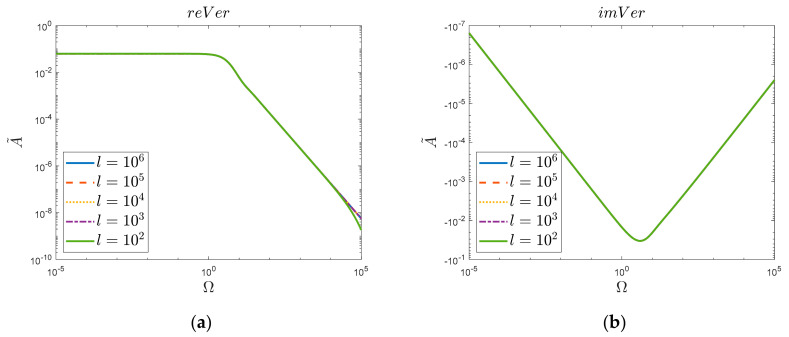
Dependence on the number of summation terms “*l*” of newly derived function: (**a**) Realis, (**b**) Imaginaris.

The above results of the simulation comparisons (Figure 3) were obtained considering one million zeros of the Bessel function (*l* = 10^6^). It was then noticed (Figure 4) that reducing the number of zeros taken into account resulted in a decrease in the convergence of the original function “*Org*” for large values of Ω. Such a decrease was not observed (Figure 5) in the case of the new obtained solution “*Ver*”; here, in the analyzed range, i.e., up to the frequency $\Omega =10^{5}$, the acceptable convergence was obtained (reVer graph, Figure 5a), taking into account only a thousand zeros (*l* = 10^3^). This fact is undoubtedly influenced by the Calogero dependency.

The obtained solution in the time domain for *k* = 2 is the basis for finding a solution for *k* = 3. According to the formula in Equation (25), we should calculate the following convolution:

(40) $$B=\int_{0}^{t}\left(u\sum_{i=1}^{\infty }e^{-\mu_{i}^{2}\frac{\nu }{R^{2}}u}+\frac{R^{2}}{\nu }\sum_{i=1}^{\infty }\frac{e^{-\mu_{i}^{2}\frac{\nu }{R^{2}}u}}{\mu_{i}^{2}}\right)\sum_{j=1}^{\infty }e^{-\mu_{j}^{2}\frac{\nu }{R^{2}}\left(t-u\right)}du.\;$$

The above notation means that we need to know the analytical solutions of the following integral:

(41) $$B=\int_{0}^{t}\left(u\sum_{i=1}^{\infty }e^{-au}+\sum_{i=1}^{\infty }\frac{e^{-au}}{a}\right)\overset{\infty }{\underset{j=1}{\sum }}e^{-b\left(t-u\right)}du.\;$$

Consequently, it means determining the solutions of the following two convolution integrals:

(42) $$B^{*}=B_{I}^{*}+B_{II}^{*}=\int_{0}^{t}ue^{-au}e^{-b\left(t-u\right)}du+\int_{0}^{t}\frac{e^{-au}}{a}e^{-b\left(t-u\right)}du.\;$$

The solution to the first of the above integrals, $B_{I}^{*}$, when a≠b is the function, is as follows:

(43) $$B_{1}^{*}=\frac{e^{-bt}}{{\left(a-b\right)}^{2}}-\frac{e^{-at}\left(t\left(a-b\right)+1\right)}{{\left(a-b\right)}^{2}},\;$$

Meanwhile, when a=b, the solution is as follows:

(44) $$B_{2}^{*}=\frac{t^{2}}{2}e^{-at}.$$

On the other hand, the solutions of the second integral, $B_{II}^{*}$, are as follows when a≠b:

(45) $$B_{3}^{*}=\frac{e^{-bt}-e^{-at}}{a^{2}-ab},$$

Meanwhile, when a=b, the solution is as follows:

(46) $$B_{4}^{*}=\frac{te^{-at}}{a}.$$

By considering their infinite series instead of single exponential expressions, the following solution is obtained for *k* = 3:

(47) $$B=\frac{t^{2}}{2}\sum_{i=1}^{\infty }e^{-\mu_{i}^{2}\frac{\nu }{R^{2}}t}+t\frac{R^{2}}{\nu }\sum_{i=1}^{\infty }e^{-\mu_{i}^{2}\frac{\nu }{R^{2}}t}\left[\frac{1}{\mu_{i}^{2}}-\sum_{\begin{array}{c} j=1\\ \;j\neq i \end{array}}^{\infty }\frac{1}{\mu_{i}^{2}-\mu_{j}^{2}}\right]+{\left(\frac{R^{2}}{\nu }\right)}^{2}\sum_{i=1}^{\infty }e^{-\mu_{i}^{2}\frac{\nu }{R^{2}}t}\left[\sum_{\begin{array}{c} j=1\\ \;j\neq i \end{array}}^{\infty }\frac{1}{\mu_{j}^{4}-\mu_{i}^{2}\mu_{j}^{2}}-\sum_{\begin{array}{c} j=1\\ \;j\neq i \end{array}}^{\infty }\frac{1}{\mu_{i}^{4}-\mu_{j}^{2}\mu_{i}^{2}}\right].$$

The last term in the square bracket can be written as follows:

(48) $$\sum_{\begin{array}{c} j=1\\ \;j\neq i \end{array}}^{\infty }\frac{1}{\mu_{j}^{4}-\mu_{i}^{2}\mu_{j}^{2}}-\sum_{\begin{array}{c} j=1\\ \;j\neq i \end{array}}^{\infty }\frac{1}{\mu_{i}^{4}-\mu_{j}^{2}\mu_{i}^{2}}=\sum_{\begin{array}{c} j=1\\ \;j\neq i \end{array}}^{\infty }\frac{\mu_{i}^{2}+\mu_{j}^{2}}{\mu_{i}^{2}\mu_{j}^{2}\left(\mu_{j}^{2}-\mu_{i}^{2}\right)}=\sum_{\begin{array}{c} j=1\\ \;j\neq i \end{array}}^{\infty }\frac{1}{\mu_{j}^{2}\left(\mu_{j}^{2}-\mu_{i}^{2}\right)}+\frac{1}{\mu_{i}^{2}}\sum_{\begin{array}{c} j=1\\ \;j\neq i \end{array}}^{\infty }\frac{1}{\left(\mu_{j}^{2}-\mu_{i}^{2}\right)}.$$

Then we can obtain the following:

(49) $$B=\frac{t^{2}}{2}\sum_{i=1}^{\infty }e^{-\mu_{i}^{2}\frac{\nu }{R^{2}}t}+t\frac{R^{2}}{\nu }\sum_{i=1}^{\infty }e^{-\mu_{i}^{2}\frac{\nu }{R^{2}}t}\left[\frac{1}{\mu_{i}^{2}}+\sum_{\begin{array}{c} j=1\\ \;j\neq i \end{array}}^{\infty }\frac{1}{\mu_{j}^{2}-\mu_{i}^{2}}\right]+{\left(\frac{R^{2}}{\nu }\right)}^{2}\sum_{i=1}^{\infty }e^{-\mu_{i}^{2}\frac{\nu }{R^{2}}t}\left[\sum_{\begin{array}{c} j=1\\ \;j\neq i \end{array}}^{\infty }\frac{1}{\mu_{j}^{2}\left(\mu_{j}^{2}-\mu_{i}^{2}\right)}+\frac{1}{\mu_{i}^{2}}\sum_{\begin{array}{c} j=1\\ \;j\neq i \end{array}}^{\infty }\frac{1}{\left(\mu_{j}^{2}-\mu_{i}^{2}\right)}\right].$$

Unfortunately, in the works of Calogero [60,61], as well as in the work of Ahmed–Calogero [62], one cannot find a solution for the function $\sum_{\begin{array}{c} j=1\\ j\neq i \end{array}}^{\infty }\frac{1}{\mu_{j}^{2}\left(\mu_{j}^{2}-\mu_{i}^{2}\right)}$ appearing in the equation (Equation (49)). The following solution can be deduced from knowing that:

(50) $$\frac{1}{b\left(b-a\right)}=\frac{1}{{\left(b-a\right)}^{2}}-\frac{a}{b{\left(a-b\right)}^{2}}.\;$$

In our case, more generally, we obtain the following:

(51) $$\overset{\infty }{\underset{\begin{array}{c} j=1\\ j\neq i \end{array}}{\sum }}\frac{1}{\mu_{j}^{2}\left(\mu_{j}^{2}-\mu_{i}^{2}\right)}=\overset{\infty }{\underset{\begin{array}{c} j=1\\ j\neq i \end{array}}{\sum }}\frac{1}{{\left(\mu_{j}^{2}-\mu_{i}^{2}\right)}^{2}}-\mu_{i}^{2}\overset{\infty }{\underset{\begin{array}{c} j=1\\ j\neq i \end{array}}{\sum }}\frac{1}{\mu_{j}^{2}{\left(\mu_{i}^{2}-\mu_{j}^{2}\right)}^{2}}.\;$$

Moreover, we also notice the following:

(52) $$\overset{\infty }{\underset{\begin{array}{c} j=1\\ j\neq i \end{array}}{\sum }}\frac{1}{{\left(\mu_{j}^{2}-\mu_{i}^{2}\right)}^{2}}=\overset{\infty }{\underset{\begin{array}{c} j=1\\ j\neq i \end{array}}{\sum }}\frac{1}{{\left(\mu_{i}^{2}-\mu_{j}^{2}\right)}^{2}}.\;$$

Thus, it is possible to use the formulas presented in the work by Ahmed–Calogero [62]:

(53) $$\overset{\infty }{\underset{\begin{array}{c} j=1\\ j\neq i \end{array}}{\sum }}\frac{1}{\mu_{j}^{2}{\left(\mu_{i}^{2}-\mu_{j}^{2}\right)}^{2}}=\frac{4\mu_{i}^{2}-23}{12\mu_{i}^{6}}\;\mathrm{and}\;\overset{\infty }{\underset{\begin{array}{c} j=1\\ j\neq i \end{array}}{\sum }}\frac{1}{{\left(\mu_{j}^{2}-\mu_{i}^{2}\right)}^{2}}=\frac{\mu_{i}^{2}-5}{12\mu_{i}^{4}}.\;$$

Then we obtain the following:

(54) $$\overset{\infty }{\underset{\begin{array}{c} j=1\\ j\neq i \end{array}}{\sum }}\frac{1}{\mu_{j}^{2}\left(\mu_{j}^{2}-\mu_{i}^{2}\right)}=\frac{\mu_{i}^{2}-5}{12\mu_{i}^{4}}-\frac{4\mu_{i}^{2}-23}{12\mu_{i}^{4}}=\frac{6-\mu_{i}^{2}}{4\mu_{i}^{4}}.\;$$

Having the solutions of Equations (35) and (54), we can return to Equation (49) and derive its final time domain form:

(55) $$B=\frac{t^{2}}{2}\overset{\infty }{\underset{i=1}{\sum }}e^{-\mu_{i}^{2}\frac{\nu }{R^{2}}t}+\frac{3}{2}t\frac{R^{2}}{\nu }\overset{\infty }{\underset{i=1}{\sum }}\frac{e^{-\mu_{i}^{2}\frac{\nu }{R^{2}}t}}{\mu_{i}^{2}}+{\left(\frac{R^{2}}{\nu }\right)}^{2}\overset{\infty }{\underset{i=1}{\sum }}e^{-\mu_{i}^{2}\frac{\nu }{R^{2}}t}\left(\frac{8-\mu_{i}^{2}}{4\mu_{i}^{4}}\right).\;$$

This equation reads in the Laplace domain as follows:

(56) $$ℒ\left\{B\right\}=\sum_{i=1}^{\infty }\frac{1}{{\left(\mu_{i}^{2}\frac{\nu }{R^{2}}+s\right)}^{3}}+\frac{3}{2}\left(\frac{R^{2}}{\nu }\right)\sum_{i=1}^{\infty }\frac{1}{\mu_{i}^{2}{\left(\mu_{i}^{2}\frac{\nu }{R^{2}}+s\right)}^{2}}+{\left(\frac{R^{2}}{\nu }\right)}^{2}\sum_{i=1}^{\infty }\frac{1}{\left(\mu_{i}^{2}\frac{\nu }{R^{2}}+s\right)}\left(\frac{8-\mu_{i}^{2}}{4\mu_{i}^{4}}\right).$$

By multiplying the formula in Equation (56) by ${\left(\frac{\nu }{R^{2}}\right)}^{3}$, one can obtain a dimensionless solution:

(57) $$\tilde{B}={\left(\overset{\infty }{\underset{i=1}{\sum }}\frac{1}{\mu_{i}^{2}+\hat{s}}\right)}^{3}=\overset{\infty }{\underset{i=1}{\sum }}\frac{1}{{\left(\mu_{i}^{2}+\hat{s}\right)}^{3}}+\frac{3}{2}\overset{\infty }{\underset{i=1}{\sum }}\frac{1}{\mu_{i}^{2}{\left(\mu_{i}^{2}+\hat{s}\right)}^{2}}+\overset{\infty }{\underset{i=1}{\sum }}\frac{1}{\left(\mu_{i}^{2}+\hat{s}\right)}\left(\frac{8-\mu_{i}^{2}}{4\mu_{i}^{4}}\right).\;$$

**Figure 6:**
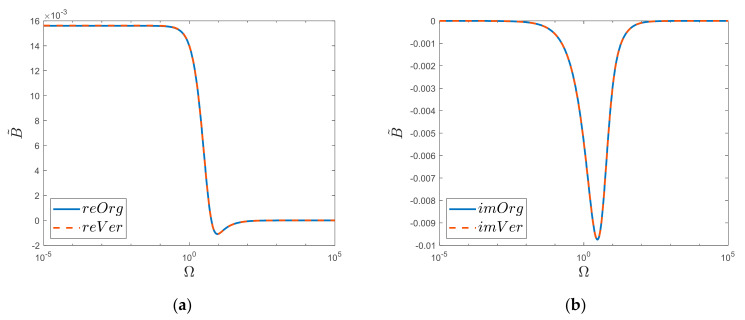
Realis (**a**) and Imaginaris (**b**) graphs of original function “*Org*” and newly derived substitute “*Ver*”. Semi−log scale for *k* = 3 case.

The comparison of the obtained solution for *k* = 3 (Equation (57)) is shown in Figure 6 below. Please note that, this time, the analyzed function is shown in semi-log graphs and not as in the case of *k* = 2 in a log-logarithmic scale. This change resulted from the nature of the variability of the analyzed solution; for *k* = 2, the real values did not exceed the abscissa axis, while for *k* = 3, such a drop occurs (see Figure 6a), hence the need for a different imaging and the use of a different ordinate axis scale.

The above comparison (Figure 6) was made by considering only one thousand zeros of the Bessel function. Further increasing the number of zeros of the Bessel functions taken into account does not increase the degree of agreement of the two functions (“*Org*” and “*Ver*”).

For *k* = 4, it is easier to apply a slightly different method than for *k* = 3. This method differs from the proposed solution from the formula in Equation (25) in that our sought solution is determined as the convolutional integral from the solutions obtained for *k* = 2:

(58) $${ℒ}^{-1}\left\{{\left(\overset{\infty }{\underset{i=1}{\sum }}\frac{1}{\mu_{i}^{2}\frac{\nu }{R^{2}}+s}\right)}^{2}{\left(\overset{\infty }{\underset{i=1}{\sum }}\frac{1}{\mu_{i}^{2}\frac{\nu }{R^{2}}+s}\right)}^{2}\right\}.\;$$

Then we obtain the following:

(59) $${ℒ}^{-1}\left\{{\left(\overset{\infty }{\underset{i=1}{\sum }}\frac{1}{\mu_{i}^{2}\frac{\nu }{R^{2}}+s}\right)}^{4}\right\}=C=\int_{0}^{t}A\left(t-u\right)A\left(u\right)du.\;$$

Remembering that the solution for *k* = 2 is described by the formula in Equation (36), we can search for solutions for *k* = 4 in the form of the following convolutional integral:

(60) $$C=\int_{0}^{t}\left(u\sum_{i=1}^{\infty }e^{-\mu_{i}^{2}\frac{\nu }{R^{2}}u}+\sum_{i=1}^{\infty }\frac{e^{-\mu_{i}^{2}\frac{\nu }{R^{2}}u}}{\mu_{i}^{2}\frac{\nu }{R^{2}}}\right)\left(\left(t-u\right)\sum_{j=1}^{\infty }e^{-\mu_{j}^{2}\frac{\nu }{R^{2}}\left(t-u\right)}+\sum_{j=1}^{\infty }\frac{e^{-\mu_{j}^{2}\frac{\nu }{R^{2}}\left(t-u\right)}}{\mu_{j}^{2}\frac{\nu }{R^{2}}}\right)du.$$

The advantage of the above approach is that the final result in the time domain will be the result of three convolutional functions (not four as if one used approach compatible with Equation (25)). It also results from the analysis of the product of solutions for *k* = 2 in the Laplace domain, by means of which the following can be shown:

(61) $${\left(\sum_{i=1}^{\infty }\frac{1}{\mu_{i}^{2}\frac{\nu }{R^{2}}+s}\right)}^{4}={\left(\sum_{i=1}^{\infty }\frac{1}{{\left(\mu_{i}^{2}\frac{\nu }{R^{2}}+s\right)}^{2}}+\frac{R^{2}}{\nu }\sum_{i=1}^{\infty }\frac{1}{\mu_{i}^{2}\left(\mu_{i}^{2}\frac{\nu }{R^{2}}+s\right)}\right)}^{2}=\sum_{i=1}^{\infty }\frac{1}{{\left(\mu_{i}^{2}\frac{\nu }{R^{2}}+s\right)}^{2}}\cdot \sum_{i=1}^{\infty }\frac{1}{{\left(\mu_{i}^{2}\frac{\nu }{R^{2}}+s\right)}^{2}}+2\sum_{i=1}^{\infty }\frac{1}{{\left(\mu_{i}^{2}\frac{\nu }{R^{2}}+s\right)}^{2}}\cdot \;\frac{R^{2}}{\nu }\sum_{i=1}^{\infty }\frac{1}{\mu_{i}^{2}\left(\mu_{i}^{2}\frac{\nu }{R^{2}}+s\right)}+\frac{R^{2}}{\nu }\sum_{i=1}^{\infty }\frac{1}{\mu_{i}^{2}\left(\mu_{i}^{2}\frac{\nu }{R^{2}}+s\right)}\cdot \frac{R^{2}}{\nu }\sum_{i=1}^{\infty }\frac{1}{\mu_{i}^{2}\left(\mu_{i}^{2}\frac{\nu }{R^{2}}+s\right)}.$$

The solution (60), as well as the return of it to the time domain with the help of the Equation (61), results, as can be seen, in the appearance of three convolution integrals. Assuming at the beginning, as for the cases of *k* = 2 and *k* = 3, the consideration of integrals for single exponential terms, one can write the following:

(62) $$C=C_{I}^{*}+C_{II}^{*}+C_{III}^{*}=\int_{0}^{t}ue^{-au}\cdot \left(t-u\right)e^{-b\left(t-u\right)}du+2\int_{0}^{t}ue^{-au}\cdot \frac{e^{-b\left(t-u\right)}}{b}du+\int_{0}^{t}\frac{e^{-au}}{a}\cdot \frac{e^{-b\left(t-u\right)}}{b}du,$$

where $a=\mu_{i}^{2}\frac{\nu }{R^{2}}$, and $b=\mu_{j}^{2}\frac{\nu }{R^{2}}$.

The solutions for these integrals are as follows:

(a) $C_{I}^{*}$ for a≠b

(63) $$C_{1}^{*}=e^{-bt}\frac{\left(t\left(a-b\right)-2\right)}{{\left(a-b\right)}^{3}}+e^{-at}\frac{\left(t\left(a-b\right)+2\right)}{{\left(a-b\right)}^{3}},\;$$

(b) $C_{I}^{*}$ for a=b

(64) $$C_{2}^{*}=\frac{1}{6}t^{3}e^{-at},\;$$

(c) $C_{II}^{*}$ for a≠b

(65) $$C_{3}^{*}=2\left(\frac{e^{-bt}}{b{\left(a-b\right)}^{2}}-e^{-at}\frac{\left(t\left(a-b\right)+1\right)}{b{\left(a-b\right)}^{2}}\right),\;$$

(d) $C_{II}^{*}$ for a=b

(66) $$C_{4}^{*}=\frac{t^{2}}{a}e^{-at},\;$$

(e) $C_{III}^{*}$ for a≠b

(67) $$C_{5}^{*}=\frac{e^{-bt}}{ab\left(a-b\right)}-\frac{e^{-at}}{ab\left(a-b\right)},$$

(f) $C_{III}^{*}$ for a=b

(68) $$C_{6}^{*}=\frac{te^{-at}}{a^{2}}.\;$$

By considering their infinite series instead of single exponential expressions, the following solution is obtained for *k* = 4:

(69) $$C=\frac{t^{3}}{6}\sum_{i=1}^{\infty }e^{-\mu_{i}^{2}\frac{\nu }{R^{2}}t}+t^{2}\frac{R^{2}}{\nu }\sum_{i=1}^{\infty }\frac{e^{-\mu_{i}^{2}\frac{\nu }{R^{2}}t}}{\mu_{i}^{2}}+t{\left(\frac{R^{2}}{\nu }\right)}^{2}\sum_{i=1}^{\infty }e^{-\mu_{i}^{2}\frac{\nu }{R^{2}}t}\left(\frac{1}{\mu_{i}^{4}}+2\sum_{\begin{array}{c} j=1\\ j\neq i \end{array}}^{\infty }\frac{1}{{\left(\mu_{i}^{2}-\mu_{j}^{2}\right)}^{2}}-2\sum_{\begin{array}{c} j=1\\ j\neq i \end{array}}^{\infty }\frac{1}{\mu_{j}^{2}\left(\mu_{i}^{2}-\mu_{j}^{2}\right)}\right)+\;4{\left(\frac{R^{2}}{\nu }\right)}^{3}\sum_{i=1}^{\infty }e^{-\mu_{i}^{2}\frac{\nu }{R^{2}}t}\left(\sum_{\begin{array}{c} j=1\\ j\neq i \end{array}}^{\infty }\frac{1}{{\left(\mu_{i}^{2}-\mu_{j}^{2}\right)}^{3}}-\sum_{\begin{array}{c} j=1\\ j\neq i \end{array}}^{\infty }\frac{1}{\mu_{i}^{2}\mu_{j}^{2}\left(\mu_{i}^{2}-\mu_{j}^{2}\right)}\right),$$

Using the Calogero [61] formulas (Equations (A5) and (A8)) collected in Appendix A of this paper and the formula in Equation (54), one can simplify the obtained equation in its final form (detailed mathematical steps are summarized in Appendix C Equation (A20)):

(70) $$C=\frac{t^{3}}{6}\sum_{i=1}^{\infty }e^{-\mu_{i}^{2}\frac{\nu }{R^{2}}t}+t^{2}\frac{R^{2}}{\nu }\sum_{i=1}^{\infty }\frac{e^{-\mu_{i}^{2}\frac{\nu }{R^{2}}t}}{\mu_{i}^{2}}+t{\left(\frac{R^{2}}{\nu }\right)}^{2}\sum_{i=1}^{\infty }e^{-\mu_{i}^{2}\frac{\nu }{R^{2}}t}\left(\frac{19-2\mu_{i}^{2}}{6\mu_{i}^{4}}\right)+{\left(\frac{R^{2}}{\nu }\right)}^{3}\sum_{i=1}^{\infty }e^{-\mu_{i}^{2}\frac{\nu }{R^{2}}t}\left(\frac{-3\mu_{i}^{2}+18}{4\mu_{i}^{6}}\right),$$

The Laplace transform of the above solution gives the following result:

(71) $$L\left\{C\right\}=\sum_{i=1}^{\infty }\frac{1}{{\left(\mu_{i}^{2}\frac{\nu }{R^{2}}+s\right)}^{4}}+2\frac{R^{2}}{\nu }\sum_{i=1}^{\infty }\frac{1}{\mu_{i}^{2}}\frac{1}{{\left(\mu_{i}^{2}\frac{\nu }{R^{2}}+s\right)}^{3}}+{\left(\frac{R^{2}}{\nu }\right)}^{2}\sum_{i=1}^{\infty }\frac{1}{{\left(\mu_{i}^{2}\frac{\nu }{R^{2}}+s\right)}^{2}}\left(\frac{19-2\mu_{i}^{2}}{6\mu_{i}^{4}}\right)+{\left(\frac{R^{2}}{\nu }\right)}^{3}\sum_{i=1}^{\infty }\frac{1}{\left(\mu_{i}^{2}\frac{\nu }{R^{2}}+s\right)}\left(\frac{18-3\mu_{i}^{2}}{4\mu_{i}^{6}}\right)=\;{\left(\sum_{i=1}^{\infty }\frac{1}{\mu_{i}^{2}\frac{\nu }{R^{2}}+s}\right)}^{4},$$

Multiplying the obtained formula by ${\left(\frac{\nu }{R^{2}}\right)}^{4}$ makes it possible to write this solution in a dimensionless form:

(72) $$\tilde{C}={\left(\sum_{i=1}^{\infty }\frac{1}{\mu_{i}^{2}+\hat{s}}\right)}^{4}=\sum_{i=1}^{\infty }\frac{1}{{\left(\mu_{i}^{2}+\hat{s}\right)}^{4}}+2\sum_{i=1}^{\infty }\frac{1}{\mu_{i}^{2}{\left(\mu_{i}^{2}+\hat{s}\right)}^{3}}+\sum_{i=1}^{\infty }\frac{1}{{\left(\mu_{i}^{2}+\hat{s}\right)}^{2}}\left(\frac{19-2\mu_{i}^{2}}{6\mu_{i}^{4}}\right)+\sum_{i=1}^{\infty }\frac{1}{\left(\mu_{i}^{2}+\hat{s}\right)}\left(\frac{18-3\mu_{i}^{2}}{4\mu_{i}^{6}}\right),$$

**Figure 7:**
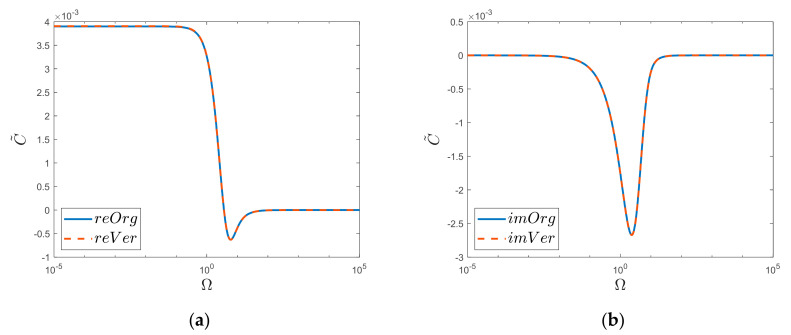
Realis (**a**) and Imaginaris (**b**) graphs of original function *“Org”* and newly derived substitute *“Ver”*. Semi−log scale for *k* = 4 case.

The comparison of the obtained dimensionless solution for *k* = 4 is shown in Figure 7. The last solution that was derived in this paper concerns the case when *k* = 5. In order to obtain this solution, the convolutional integral from the solutions for *k* = 4 and *k* = 1 was used. This solution was obtained with the help of the formula presented in Equation (25) (it is possible to obtain the same final solution as a convolution from solutions for *k* = 2 and *k* = 3). The detailed procedure of obtaining the final result is similar to the one discussed in the previous cases (*k* = 2, 3, and 4); hence, it is not discussed here again. For the sake of clarity, however, it is worth writing down (see Equation (A21) in Appendix C) all the obtained infinite series of Calogero–Ahmed type from which the final coefficients of the last terms were determined.

It is possible that such knowledge will enable us to propose a general solution in the near future. In order to obtain the final result (and in particular the coefficient for the last term), it was necessary to apply a number of formulas for infinite series of the Calogero–type (Equations (2), (4), (5) and (14)–(18) from Reference [62], for which the values are summarized in Appendix A (in our case for *h* = 0)). Finally, the following result was obtained:

(73) $$D=\frac{t^{4}}{24}\sum_{i=1}^{\infty }e^{-\mu_{i}^{2}\frac{\nu }{R^{2}}t}+\frac{t^{3}}{6}\frac{R^{2}}{\nu }\sum_{i=1}^{\infty }e^{-\mu_{i}^{2}\frac{\nu }{R^{2}}t}\left(\frac{5}{2\mu_{i}^{2}}\right)+\frac{t^{2}}{2}{\left(\frac{R^{2}}{\nu }\right)}^{2}\sum_{i=1}^{\infty }e^{-\mu_{i}^{2}\frac{\nu }{R^{2}}t}\left(\frac{55-5\mu_{i}^{2}}{12\mu_{i}^{4}}\right)+\;t{\left(\frac{R^{2}}{\nu }\right)}^{3}\sum_{i=1}^{\infty }e^{-\mu_{i}^{2}\frac{\nu }{R^{2}}t}\left(\frac{350-55\mu_{i}^{2}}{48\mu_{i}^{6}}\right)+{\left(\frac{R^{2}}{\nu }\right)}^{4}\sum_{i=1}^{\infty }e^{-\mu_{i}^{2}\frac{\nu }{R^{2}}t}\left(\frac{512-106\mu_{i}^{2}+3\mu_{i}^{4}}{48\mu_{i}^{8}}\right),$$

Moreover, in the Laplace domain, we obtain the following:

(74) $$L\left\{D\right\}=\sum_{i=1}^{\infty }\frac{1}{{\left(\mu_{i}^{2}\frac{\nu }{R^{2}}+s\right)}^{5}}+\frac{R^{2}}{\nu }\sum_{i=1}^{\infty }\frac{1}{{\left(\mu_{i}^{2}\frac{\nu }{R^{2}}+s\right)}^{4}}\left(\frac{5}{2\mu_{i}^{2}}\right)+{\left(\frac{R^{2}}{\nu }\right)}^{2}\sum_{i=1}^{\infty }\frac{1}{{\left(\mu_{i}^{2}\frac{\nu }{R^{2}}+s\right)}^{3}}\left(\frac{55-5\mu_{i}^{2}}{12\mu_{i}^{4}}\right)+\;{\left(\frac{R^{2}}{\nu }\right)}^{3}\sum_{i=1}^{\infty }\frac{1}{{\left(\mu_{i}^{2}\frac{\nu }{R^{2}}+s\right)}^{2}}\left(\frac{350-55\mu_{i}^{2}}{48\mu_{i}^{6}}\right)+{\left(\frac{R^{2}}{\nu }\right)}^{4}\sum_{i=1}^{\infty }\frac{1}{\left(\mu_{i}^{2}\frac{\nu }{R^{2}}+s\right)}\left(\frac{512-106\mu_{i}^{2}+3\mu_{i}^{4}}{48\mu_{i}^{8}}\right)={\left(\sum_{i=1}^{\infty }\frac{1}{\mu_{i}^{2}\frac{\nu }{R^{2}}+s}\right)}^{5},$$

Multiplying the obtained formula by ${\left(\frac{\nu }{R^{2}}\right)}^{5}$ makes it possible to write this solution in a dimensionless form:

(75) $$\tilde{D}={\left(\sum_{i=1}^{\infty }\frac{1}{\mu_{i}^{2}+\hat{s}}\right)}^{5}=\sum_{i=1}^{\infty }\frac{1}{{\left(\mu_{i}^{2}+\hat{s}\right)}^{5}}+\frac{5}{2}\sum_{i=1}^{\infty }\frac{1}{\mu_{i}^{2}{\left(\mu_{i}^{2}+\hat{s}\right)}^{4}}+\sum_{i=1}^{\infty }\frac{1}{{\left(\mu_{i}^{2}+\hat{s}\right)}^{3}}\left(\frac{55-5\mu_{i}^{2}}{12\mu_{i}^{4}}\right)+\;\sum_{i=1}^{\infty }\frac{1}{{\left(\mu_{i}^{2}+\hat{s}\right)}^{2}}\left(\frac{350-55\mu_{i}^{2}}{48\mu_{i}^{6}}\right)+\sum_{i=1}^{\infty }\frac{1}{\left(\mu_{i}^{2}+\hat{s}\right)}\left(\frac{512-106\mu_{i}^{2}+3\mu_{i}^{4}}{48\mu_{i}^{8}}\right),$$

**Figure 8:**
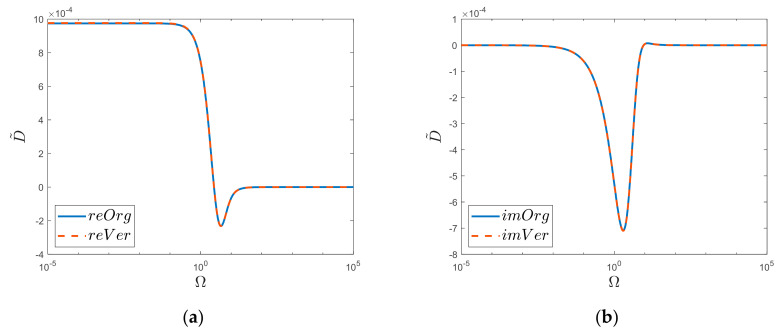
Realis (**a**) and Imaginaris (**b**) graphs of original function “*Org*” and newly derived substitute “*Ver*”. Semi−log scale for *k* = 5 case.

The comparison of the obtained dimensionless solution for *k* = 5 is shown in Figure 8. The further determination of successive solutions for powers greater than 5 becomes problematic in terms of manual implementation, which is associated with the need to use more and more complex Calogero–Ahmad series solutions. These solutions are the result of successive convolutions. In addition, the literature lacks solutions to these series, so it becomes necessary to constantly determine new formulas or find a relationship that would enable the analytical determination of their mathematical forms.

The most important advantage of the presented novel technique is the possibility to fully analytically describe the dynamic viscosity function in an extended time range. The inverse Laplace transform of this important function was developed for the first time (to the best knowledge of the authors). With its help, it will be possible to find a complete solution to water hammer equations (investigations are in progress to achieve this goal) and to define accurate approximation solutions of these phenomena (traditionally preferred by practitioners due to their mathematical simplicity). Another advantage of the presented technique is that it can be easily extended (solution for larger *k* can be found) with the help of a machine-learning technique. Initial work is currently underway on the implementation of such a computational algorithm.

## 5. Comparison of *M* Function Transform in Time and Frequency Domain

The knowledge of the convolutional solutions for the first power expansions of the analyzed dynamic viscosity function allows us to present a collective solution. Looking at the constants that appear in the new notation of the analyzed function (double infinite summation form, Equation (20)), it can be seen that they are based on the values of the gamma function that can be expressed as a definite integral for ℜ(a)>0 in Euler’s integral form [74]:

(76) $$\Gamma \left(a\right)=\int_{0}^{\infty }t^{a-1}e^{-t}dt.$$

A time domain solution of such rational equations, i.e.,

(77) $$\sum_{k=0}^{\infty }{\left(\sum_{i=1}^{\infty }\frac{1}{\mu_{i}^{2}\frac{\nu }{R^{2}}+s}\right)}^{k},$$

was found in the previous section as an inverse Laplace transform for the first five powers of *k* (for collected successive solutions of this type, see Appendix B).

Knowing the above solutions and the values of the constants $\frac{\Gamma \left(k+0.5\right)}{\Gamma \left(k+1\right)}\cdot \frac{4^{k}}{\sqrt{\pi }}$ calculated for the first five powers of *k*, we are able to write the final form of the dynamic viscosity function in time domain, i.e., the inverse Laplace transform of our searched function:

(78) $$L^{-1}\left\{\sqrt{\frac{I_{0}\left(\sqrt{s\frac{R^{2}}{\nu }}\right)}{I_{2}\left(\sqrt{s\frac{R^{2}}{\nu }}\right)}}\right\}=\delta \left(t\right)+2\frac{\nu }{R^{2}}\sum_{i=1}^{\infty }e^{-\mu_{i}^{2}\hat{t}}+6\frac{\nu }{R^{2}}\left[\hat{t}\sum_{i=1}^{\infty }e^{-\mu_{i}^{2}\hat{t}}+\sum_{i=1}^{\infty }\frac{e^{-\mu_{i}^{2}\hat{t}}}{\mu_{i}^{2}}\right]+\;20\frac{\nu }{R^{2}}\left[\frac{{\hat{t}}^{2}}{2}\sum_{i=1}^{\infty }e^{-\mu_{i}^{2}\hat{t}}+\frac{3\hat{t}}{2}\sum_{i=1}^{\infty }\frac{e^{-\mu_{i}^{2}\hat{t}}}{\mu_{i}^{2}}+\sum_{i=1}^{\infty }e^{-\mu_{i}^{2}\hat{t}}\left(\frac{8-\mu_{i}^{2}}{4\mu_{i}^{4}}\right)\right]+70\frac{\nu }{R^{2}}[\frac{{\hat{t}}^{3}}{6}\sum_{i=1}^{\infty }e^{-\mu_{i}^{2}\hat{t}}+\;{\hat{t}}^{2}\sum_{i=1}^{\infty }\frac{e^{-\mu_{i}^{2}\hat{t}}}{\mu_{i}^{2}}+\hat{t}\sum_{i=1}^{\infty }e^{-\mu_{i}^{2}\hat{t}}\left(\frac{19-2\mu_{i}^{2}}{6\mu_{i}^{4}}\right)+\sum_{i=1}^{\infty }e^{-\mu_{i}^{2}\hat{t}}\left(\frac{18-3\mu_{i}^{2}}{4\mu_{i}^{6}}\right)]+252\frac{\nu }{R^{2}}[\frac{{\hat{t}}^{4}}{24}\sum_{i=1}^{\infty }e^{-\mu_{i}^{2}\hat{t}}+\;\frac{5{\hat{t}}^{3}}{12}\sum_{i=1}^{\infty }\frac{e^{-\mu_{i}^{2}\hat{t}}}{\mu_{i}^{2}}+\frac{{\hat{t}}^{2}}{2}\sum_{i=1}^{\infty }e^{-\mu_{i}^{2}\hat{t}}\left(\frac{55-5\mu_{i}^{2}}{12\mu_{i}^{4}}\right)+\hat{t}\sum_{i=1}^{\infty }e^{-\mu_{i}^{2}\hat{t}}\left(\frac{350-55\mu_{i}^{2}}{48\mu_{i}^{6}}\right)+\;\sum_{i=1}^{\infty }e^{-\mu_{i}^{2}\hat{t}}\left(\frac{512-106\mu_{i}^{2}+3\mu_{i}^{4}}{48\mu_{i}^{8}}\right)],$$

Interestingly, the above sum can also be written in a different (shortened) form by ordering the coefficients with appropriate sums:

(79) $$L^{-1}\left\{\sqrt{\frac{I_{0}\left(\sqrt{s\frac{R^{2}}{\nu }}\right)}{I_{2}\left(\sqrt{s\frac{R^{2}}{\nu }}\right)}}\right\}=\frac{\nu }{R^{2}}[\delta \left(t\right)\frac{R^{2}}{\nu }+\left(2+6\hat{t}+10{\hat{t}}^{2}+\frac{35}{3}{\hat{t}}^{3}+\frac{21}{2}{\hat{t}}^{4}\right)\sum_{i=1}^{\infty }e^{-\mu_{i}^{2}\hat{t}}+\;\left(1+\frac{20}{3}\hat{t}+\frac{35}{2}{\hat{t}}^{2}+105{\hat{t}}^{3}\right)\sum_{i=1}^{\infty }\frac{e^{-\mu_{i}^{2}\hat{t}}}{\mu_{i}^{2}}+\left(\frac{13}{4}-\frac{805}{12}\hat{t}+\frac{1155}{2}{\hat{t}}^{2}\right)\sum_{i=1}^{\infty }\frac{e^{-\mu_{i}^{2}\hat{t}}}{\mu_{i}^{4}}+(-\frac{483}{2}+\;\frac{3675}{2}\hat{t})\sum_{i=1}^{\infty }\frac{e^{-\mu_{i}^{2}\hat{t}}}{\mu_{i}^{6}}+2688\sum_{i=1}^{\infty }\frac{e^{-\mu_{i}^{2}\hat{t}}}{\mu_{i}^{8}}],$$

Let us compare our obtained solution of the inverse transform of the dynamic viscosity function, *M*, with the inverse Laplace transform of the quasi-steady form of this function, *M_q-s_*, i.e., with the inverse transform of the function described by the formula in Equation (13):

(80) $${ℒ}^{-1}\left\{M_{q-s}=\sqrt{1+\frac{8}{s}\frac{\nu }{R^{2}}}\right\}=4\frac{\nu }{R^{2}}e^{-4\frac{\nu }{R^{2}}t}\left(I_{0}\left(4\frac{\nu }{R^{2}}t\right)+I_{1}\left(4\frac{\nu }{R^{2}}t\right)\right)+\delta \left(t\right).$$

Now we are going to investigate asymptotic solutions, as well. With the help of the generalized Puiseux series, one can find a series expansion of the dynamic viscosity function, *M* (Equation (9)), for small $\hat{s}\to 0$:

(81) $$M_{\hat{s}=small}\approx \sqrt{\frac{8}{\hat{s}}}+\frac{\sqrt{\hat{s}}}{3\sqrt{2}}-\frac{\sqrt{{\hat{s}}^{3}}}{64\sqrt{2}}+\frac{47\sqrt{{\hat{s}}^{5}}}{34,560\sqrt{2}}=\frac{\left(\hat{s}\left(\hat{s}\left(47\hat{s}-540\right)+11,520\right)+138,240\right)}{34,560\sqrt{2}\sqrt{\hat{s}}}.$$

An inverse Laplace transform of the above function gives an approximation in the time domain that is valid for relatively large dimensionless times:

(82) $$m_{\hat{t}=large}\approx \frac{\nu }{R^{2}}\left[\sqrt{\frac{8}{\pi \hat{t}}}-\frac{1}{6\sqrt{2\pi }{\hat{t}}^{\frac{3}{2}}}-\frac{3}{256\sqrt{2\pi }{\hat{t}}^{\frac{5}{2}}}-\frac{47}{18,432\sqrt{2\pi }{\hat{t}}^{\frac{7}{2}}}\right]ℍ\left(t\right),$$

where ℍ(t) is a Heaviside step function, and $\hat{t}=t\frac{\nu }{R^{2}}$.

Moreover, for large $\hat{s}\to \infty$, one can expand the *M* function by using the Hankel asymptotic expansion of the modified Bessel functions (p. 377 in Reference [64]):

(83) $$I_{p}\left(z\right)=\frac{e^{z}}{\sqrt{2\pi z}}\left[1-\frac{4p^{2}-1}{8z}+\frac{\left(4p^{2}-1\right)\left(4p^{2}-9\right)}{2!{\left(8z\right)}^{2}}-\frac{\left(4p^{2}-1\right)\left(4p^{2}-9\right)\left(4p^{2}-25\right)}{3!{\left(8z\right)}^{3}}+\ldots \right]\;\;\left(\left|arg\;z\right|<\pi /2\right).$$

The use of the above expansion in our case results in the following:

(84) $$\sqrt{\frac{I_{0}\left(z\right)}{I_{2}\left(z\right)}}=\sqrt{\frac{1+\frac{1}{8z}+\frac{9}{128z^{2}}+\frac{75}{1024z^{3}}}{1-\frac{15}{8z}+\frac{105}{128z^{2}}+\frac{315}{1024z^{3}}}}.$$

The series expansion for z=∞ finally gives the following:

(85) $$\sqrt{\frac{I_{0}\left(z\right)}{I_{2}\left(z\right)}}=1+\frac{1}{z}+\frac{1}{z^{2}}+\frac{7}{8z^{3}}+\frac{617}{1024z^{4}}.$$

The above expansion is similar to the one presented by Holmboe [33] and by Brown and Nelson [75]. Substituting for $z=\sqrt{\hat{s}}$, one obtains the following:

(86) $$M_{\hat{s}=large}\approx 1+\frac{1}{\sqrt{\hat{s}}}+\frac{1}{\hat{s}}+\frac{7}{8\sqrt{{\hat{s}}^{3}}}.$$

The inverse Laplace transform of the above solution gives an approximate form (correct for relatively small dimensionless times) of the dynamic viscosity function in the time domain:

(87) $$m_{\hat{t}=small}\approx \frac{\nu }{R^{2}}\left[\delta \left(t\right)\frac{R^{2}}{\nu }+\frac{1}{\sqrt{\pi \hat{t}}}+1+\frac{7}{4}\sqrt{\frac{\hat{t}}{\pi }}\right].$$

**Figure 9:**
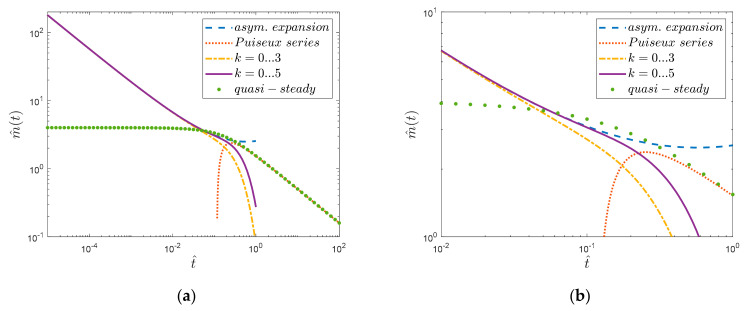
Comparison of novel solution received for initial power terms in time domain: (**a**) Extended range, (**b**) Enlarge.

**Figure 10:**
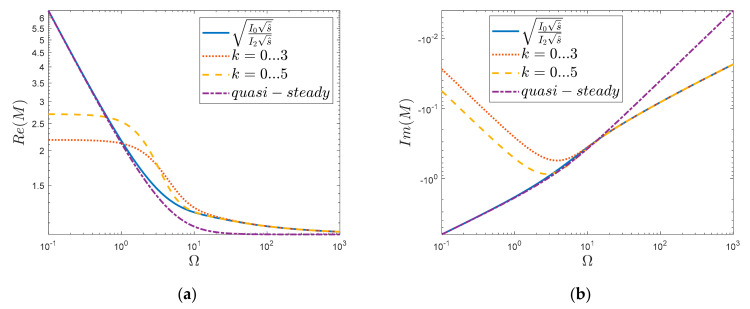
Comparison of novel solution received for initial power terms in the frequency domain: (**a**) Realis graph, (**b**) Imaginaris graph.

In order to emphasize the influence of the number of power expansion terms on the fit of the obtained results, as presented in Figure 9 and Figure 10, a comparison is made here for two cases of analytical solutions, i.e., for *k* = 0 … 3 and *k* = 0 … 5. Figure 9 shows a comparison of the histories obtained in the time domain (discussed briefly above). Meanwhile, in Figure 10, the solutions for *k* = 0 … 3 and *k* = 0 … 5 are compared with the output function (Equation (9)) and its quasi-steady counterpart (Equation (13)). Figure 9 compares the solutions in dimensionless form, i.e., $\hat{m}=m\frac{R^{2}}{\nu }$.

The presented comparisons show that the use of the analytical solution based only on the first three expansions of power *k* = 0 … 3 is not sufficient to describe the analyzed dynamic function. The approximate form of the solution in the time domain, which is based on the first five power expansions *k* = 0 … 5, gives a much better fit. It seems that the next five exponential terms should guarantee a smooth transition in the time domain to the solution that is the inverse transformation of the dynamic viscosity function expansion into the Puiseux series (red dotted line). Knowledge of this transition will allow us to approximate a complex analytical solution and to propose a simplified analytical solution of water hammer. The presented comparison shows that the inverse Laplace transform with asymptotic expansion (blue line of line graph) is suitable for use in the dimensionless time domain in the range of $0<\hat{t}<10^{-1}$. For dimensionless times $\hat{t}\geq 1$, this function can also be correctly modelled with the help of the quasi-steady solution (line of large green dots). The transitional range of this function ($10^{-1}\leq \hat{t}\leq 10^{0}$), in which significant dynamics of its variability are observed, is the range that is responsible for the water hammer in oil–hydraulic systems (0.05≤Wh≤0.5). Hence, it is important to further extend the analytical solution proposed in this paper.

The obtained analytical solutions based on the initial power expansions *k* = 0 … 3 and *k* = 0 … 5 were also compared with the exact solution of the analyzed function in the frequency domain (Figure 10). Both the comparison of the real part (Figure 10a) and the imaginary part (Figure 10b) show that successive expansions extend the compatibility range of the new solution. Therefore, it is worth extending the analyzed analytical function (by successive powers of *k*) so that it represents a high accuracy in the range of dimensionless frequencies significant from the practical point of view, i.e., $10^{0}\leq \Omega \leq 10^{1}$.

## 6. Conclusions

The novel solutions presented in this paper are an important first step in the development of a complete analytical solution of water hammer equations; however, we consider them to be worthy of sharing with a wider group of researchers. A complete analytical solution can be obtained with the help of the approach presented in this paper through machine learning, as well as with the help of artificial intelligence (AI). To develop such solutions, it is necessary to generalize the series based on the zeros of Bessel functions of the Calogero–Ahmed type. To this day, we know too little about these infinite series. Their polynomial solutions for higher power expansion of analyzed dynamic viscosity function looks to be the key point to completely describe the theory of such Laplace inverses. The extended final solution that will be obtained in the future can be approximated by finite exponential series, exactly those that are currently used in weighting functions for effective numerical calculations of the wall shear stresses in unsteady pipe flows.

Further investigations aimed at finding the final simplified solution to the problem analyzed by the authors in this paper are underway. We should learn whether the solutions presented in recent works in the field of mathematical physics that were kindly proposed by the reviewers would help in the development of the simplified analytical solution of unsteady pipe-flow equations [76,77,78,79]. The initial attempt to find/formulate a generalized analytical formula for water hammer equations in the time domain by using the dynamical viscosity function shows that such a solution will probably be similar in form to the differential form of describing the fractional Bessel functions, as well as a derivative equation that describe the Laguerre polynomials (see Equation (25) in page 319 in Doetsch’s book [80]). However, the missing important part will be the definition of new polynomials that appear during calculation (marked red terms in Appendix B of this paper).

The presented and discussed new method seems to be very promising, as it can be used not only in fluid mechanics but also in other basic sciences: mathematics, materials engineering, thermodynamics, physics, and medicine; it can also be used generally wherever there are solutions based on the similar functions discussed in this paper, i.e., exponential functions and series of the Calogero–Ahmed type.

We do hope that researchers will be able to further simplify the proposed time domain analytical solution. Perhaps they will also manage to simplify the final form, that is, to collapse our solution to the final form; in other words, they need to complete the opposite task to the one performed in Laplace domain, where we decomposed the original function into a doubly infinite series.

## Data Availability

Not applicable.

## Nomenclature

**Name**	**Description**	**Unit**
c	pressure wave speed	(m/s)
ℍ	Heaviside step function	(-)
j	unit imaginary number: $j=\sqrt{-1}$	(-)
L	pipe length	(m)
m	dynamic viscosity function in the time domain	(1/s)
M	dynamic viscosity function	(-)
p	average pressure as a function of *x* and *t*	(Pa)
$\bar{p}$	axial pressure in cylindrical coordinates—function of *x* and *t*	(Pa)
r	independent variable for radial direction in cylindrical coordinates	(m)
R	inner pipe radius	(m)
s	Laplace complex variable	(s^−1^)
$\hat{s}$	dimensionless Laplace variable	(-)
t	time	(s)
$\hat{t}$	dimensionless time	(-)
v	average velocity in cross-section	(m/s)
$\bar{v}$	axial velocity in cylindrical coordinates—function of *x*, *r*, and *t*	(m/s)
$v_{0}$	initial velocity in pre-transient flow	(m/s)
Wh	water hammer number: $Wh=\nu L/\left(cR^{2}\right)$	(-)
x	independent variable for axial direction in cylindrical coordinates	(m)
ℤ	Joukowsky pressure-rise formula: $\mathbb{Z}=\rho cv_{0}$	(Pa)
δ	Dirac delta function	(-)
*η_i_*	consecutive zeros of first-kind Bessel functions of the second order	(-)
μ	dynamic viscosity	(Pa∙s)
$\mu_{i}$	consecutive zeros of first-kind Bessel functions of the zero order	(-)
ν	kinematic viscosity	(m^2^/s)
ρ	liquid density	(kg/m^3^)
ω	frequency	(s^−1^)
Γ	gamma function	(-)
Ω	dimensionless frequency	(-)

## Appendix A

The Calogero–Ahmad series for zero-order first-kind Bessel functions (*h* = 0) used in this paper are as follows:

(A1) $$\sum_{\begin{array}{c} j=1\\ \;j\neq i \end{array}}^{\infty }\frac{1}{\mu_{i}^{2}-\mu_{j}^{2}}=-\frac{1}{2\mu_{i}^{2}}.$$

(A2) $$\sum_{\begin{array}{c} j=1\\ j\neq i \end{array}}^{\infty }\frac{1}{\mu_{j}^{2}\left(\mu_{i}^{2}-\mu_{j}^{2}\right)}=\frac{\mu_{i}^{2}-6}{4\mu_{i}^{4}}.$$

(A3) $$\sum_{\begin{array}{c} j=1\\ j\neq i \end{array}}^{\infty }\frac{1}{\mu_{j}^{4}\left(\mu_{i}^{2}-\mu_{j}^{2}\right)}=\frac{\mu_{i}^{4}+8\mu_{i}^{2}-80}{32\mu_{i}^{6}}.$$

(A4) $$\sum_{\begin{array}{c} j=1\\ j\neq i \end{array}}^{\infty }\frac{1}{\mu_{j}^{6}\left(\mu_{i}^{2}-\mu_{j}^{2}\right)}=\frac{\mu_{i}^{6}+6\mu_{i}^{4}+48\mu_{i}^{2}-672}{192\mu_{i}^{8}}.$$

(A5) $$\sum_{\begin{array}{c} j=1\\ \;j\neq i \end{array}}^{\infty }\frac{1}{{\left(\mu_{i}^{2}-\mu_{j}^{2}\right)}^{2}}=\frac{\mu_{i}^{2}-5}{12\mu_{i}^{4}}.$$

(A6) $$\sum_{\begin{array}{c} j=1\\ \;j\neq i \end{array}}^{\infty }\frac{1}{\mu_{j}^{2}{\left(\mu_{i}^{2}-\mu_{j}^{2}\right)}^{2}}=\frac{4\mu_{i}^{2}-23}{12\mu_{i}^{6}}.$$

(A7) $$\sum_{\begin{array}{c} j=1\\ \;j\neq i \end{array}}^{\infty }\frac{1}{\mu_{j}^{4}{\left(\mu_{i}^{2}-\mu_{j}^{2}\right)}^{2}}=\frac{3\mu_{i}^{4}+56\mu_{i}^{2}-424}{96\mu_{i}^{8}}.$$

(A8) $$\sum_{\begin{array}{c} j=1\\ \;j\neq i \end{array}}^{\infty }\frac{1}{{\left(\mu_{i}^{2}-\mu_{j}^{2}\right)}^{3}}=\frac{\mu_{i}^{2}-6}{16\mu_{i}^{6}}.$$

(A9) $$\sum_{\begin{array}{c} j=1\\ \;j\neq i \end{array}}^{\infty }\frac{1}{\mu_{j}^{2}{\left(\mu_{i}^{2}-\mu_{j}^{2}\right)}^{3}}=\frac{19\mu_{i}^{2}-110}{48\mu_{i}^{8}}.$$

## Appendix B

(a) Solutions in the time domain

(A10) $$k=1:\;\sum_{i=1}^{\infty }e^{-\mu_{i}^{2}\frac{\nu }{R^{2}}t}.$$

(A11) $$k=2:\;t\sum_{i=1}^{\infty }e^{-\mu_{i}^{2}\frac{\nu }{R^{2}}t}+\frac{R^{2}}{\nu }\sum_{i=1}^{\infty }\left(\frac{1}{\mu_{i}^{2}}\right)e^{-\mu_{i}^{2}\frac{\nu }{R^{2}}t}.$$

(A12) $$k=3:\;\frac{t^{2}}{2}\sum_{i=1}^{\infty }e^{-\mu_{i}^{2}\frac{\nu }{R^{2}}t}+t\frac{R^{2}}{\nu }\sum_{i=1}^{\infty }e^{-\mu_{i}^{2}\frac{\nu }{R^{2}}t}\left(\frac{3}{2\mu_{i}^{2}}\right)+{\left(\frac{R^{2}}{\nu }\right)}^{2}\sum_{i=1}^{\infty }e^{-\mu_{i}^{2}\frac{\nu }{R^{2}}t}\left(\frac{8-\mu_{i}^{2}}{4\mu_{i}^{4}}\right).$$

(A13) $$k=4:\;\frac{t^{3}}{6}\sum_{i=1}^{\infty }e^{-\mu_{i}^{2}\frac{\nu }{R^{2}}t}+\frac{t^{2}}{2}\frac{R^{2}}{\nu }\sum_{i=1}^{\infty }e^{-\mu_{i}^{2}\frac{\nu }{R^{2}}t}\left(\frac{2}{\mu_{i}^{2}}\right)+t{\left(\frac{R^{2}}{\nu }\right)}^{2}\sum_{i=1}^{\infty }e^{-\mu_{i}^{2}\frac{\nu }{R^{2}}t}\left(\frac{19-2\mu_{i}^{2}}{6\mu_{i}^{4}}\right)+{\left(\frac{R^{2}}{\nu }\right)}^{3}\sum_{i=1}^{\infty }e^{-\mu_{i}^{2}\frac{\nu }{R^{2}}t}\left(\frac{18-3\mu_{i}^{2}}{4\mu_{i}^{6}}\right).$$

(A14) $$k=5:\;\frac{t^{4}}{24}\sum_{i=1}^{\infty }e^{-\mu_{i}^{2}\frac{\nu }{R^{2}}t}+\frac{t^{3}}{6}\frac{R^{2}}{\nu }\sum_{i=1}^{\infty }e^{-\mu_{i}^{2}\frac{\nu }{R^{2}}t}\left(\frac{5}{2\mu_{i}^{2}}\right)+\frac{t^{2}}{2}{\left(\frac{R^{2}}{\nu }\right)}^{2}\sum_{i=1}^{\infty }e^{-\mu_{i}^{2}\frac{\nu }{R^{2}}t}\left(\frac{55-5\mu_{i}^{2}}{12\mu_{i}^{4}}\right)+\;t{\left(\frac{R^{2}}{\nu }\right)}^{3}\sum_{i=1}^{\infty }e^{-\mu_{i}^{2}\frac{\nu }{R^{2}}t}\left(\frac{350-55\mu_{i}^{2}}{48\mu_{i}^{6}}\right)+{\left(\frac{R^{2}}{\nu }\right)}^{4}\sum_{i=1}^{\infty }e^{-\mu_{i}^{2}\frac{\nu }{R^{2}}t}\left(\frac{512-106\mu_{i}^{2}+3\mu_{i}^{4}}{48\mu_{i}^{8}}\right).$$

(b) Solutions in the Laplace domain

(A15) $$k=1:\;\sum_{i=1}^{\infty }\frac{1}{\mu_{i}^{2}\frac{\nu }{R^{2}}+s}.$$

(A16) $$k=2:\;\sum_{i=1}^{\infty }\frac{1}{{\left(\mu_{i}^{2}\frac{\nu }{R^{2}}+s\right)}^{2}}+\frac{R^{2}}{\nu }\sum_{i=1}^{\infty }\left(\frac{1}{\mu_{i}^{2}}\right)\frac{1}{\left(\mu_{i}^{2}\frac{\nu }{R^{2}}+s\right)}={\left(\sum_{i=1}^{\infty }\frac{1}{\mu_{i}^{2}\frac{\nu }{R^{2}}+s}\right)}^{2}.$$

(A17) $$k=3:\;\sum_{i=1}^{\infty }\frac{1}{{\left(\mu_{i}^{2}\frac{\nu }{R^{2}}+s\right)}^{3}}+\frac{R^{2}}{\nu }\sum_{i=1}^{\infty }\frac{1}{{\left(\mu_{i}^{2}\frac{\nu }{R^{2}}+s\right)}^{2}}\left(\frac{3}{2\mu_{i}^{2}}\right)+{\left(\frac{R^{2}}{\nu }\right)}^{2}\sum_{i=1}^{\infty }\frac{1}{\left(\mu_{i}^{2}\frac{\nu }{R^{2}}+s\right)}\left(\frac{8-\mu_{i}^{2}}{4\mu_{i}^{4}}\right)={\left(\sum_{i=1}^{\infty }\frac{1}{\mu_{i}^{2}\frac{\nu }{R^{2}}+s}\right)}^{3}.$$

(A18) $$k=4:\;\sum_{i=1}^{\infty }\frac{1}{{\left(\mu_{i}^{2}\frac{\nu }{R^{2}}+s\right)}^{4}}+\frac{R^{2}}{\nu }\sum_{i=1}^{\infty }\frac{1}{{\left(\mu_{i}^{2}\frac{\nu }{R^{2}}+s\right)}^{3}}\left(\frac{2}{\mu_{i}^{2}}\right)+{\left(\frac{R^{2}}{\nu }\right)}^{2}\sum_{i=1}^{\infty }\frac{1}{{\left(\mu_{i}^{2}\frac{\nu }{R^{2}}+s\right)}^{2}}\left(\frac{19-2\mu_{i}^{2}}{6\mu_{i}^{4}}\right)+{\left(\frac{R^{2}}{\nu }\right)}^{3}\sum_{i=1}^{\infty }\frac{1}{\left(\mu_{i}^{2}\frac{\nu }{R^{2}}+s\right)}\left(\frac{18-3\mu_{i}^{2}}{4\mu_{i}^{6}}\right)=\;{\left(\sum_{i=1}^{\infty }\frac{1}{\mu_{i}^{2}\frac{\nu }{R^{2}}+s}\right)}^{4}.$$

(A19) $$k=5:\;\sum_{i=1}^{\infty }\frac{1}{{\left(\mu_{i}^{2}\frac{\nu }{R^{2}}+s\right)}^{5}}+\frac{R^{2}}{\nu }\sum_{i=1}^{\infty }\frac{1}{{\left(\mu_{i}^{2}\frac{\nu }{R^{2}}+s\right)}^{4}}\left(\frac{5}{2\mu_{i}^{2}}\right)+{\left(\frac{R^{2}}{\nu }\right)}^{2}\sum_{i=1}^{\infty }\frac{1}{{\left(\mu_{i}^{2}\frac{\nu }{R^{2}}+s\right)}^{3}}\left(\frac{55-5\mu_{i}^{2}}{12\mu_{i}^{4}}\right)+\;{\left(\frac{R^{2}}{\nu }\right)}^{3}\sum_{i=1}^{\infty }\frac{1}{{\left(\mu_{i}^{2}\frac{\nu }{R^{2}}+s\right)}^{2}}\left(\frac{350-55\mu_{i}^{2}}{48\mu_{i}^{6}}\right)+{\left(\frac{R^{2}}{\nu }\right)}^{4}\sum_{i=1}^{\infty }\frac{1}{\left(\mu_{i}^{2}\frac{\nu }{R^{2}}+s\right)}\left(\frac{512-106\mu_{i}^{2}+3\mu_{i}^{4}}{48\mu_{i}^{8}}\right)={\left(\sum_{i=1}^{\infty }\frac{1}{\mu_{i}^{2}\frac{\nu }{R^{2}}+s}\right)}^{5}.$$

In the above solutions the values of final coefficients were found by using the Calogero–Ahmed identities [61,62,63] discussed in these papers. The coefficients highlighted in the red color form unknown polynomial series. If completely described, they should be termed as Calogero–Ahmed polynomials.

## Appendix C

This appendix shows the intermediate forms of the selected solutions. The purpose of showing them is to give the reader an insight into how to find a mathematical formula for the higher powers of the presented analytical solution:

(A20) $$C=\frac{t^{3}}{6}\sum_{i=1}^{\infty }e^{-\mu_{i}^{2}\frac{\nu }{R^{2}}t}+t^{2}\frac{R^{2}}{\nu }\sum_{i=1}^{\infty }\frac{e^{-\mu_{i}^{2}\frac{\nu }{R^{2}}t}}{\mu_{i}^{2}}+t{\left(\frac{R^{2}}{\nu }\right)}^{2}\sum_{i=1}^{\infty }e^{-\mu_{i}^{2}\frac{\nu }{R^{2}}t}\left(\frac{1}{\mu_{i}^{4}}+2\frac{\mu_{i}^{2}-5}{12\mu_{i}^{4}}-2\frac{3\mu_{i}^{2}-18}{12\mu_{i}^{4}}\right)+\;4{\left(\frac{R^{2}}{\nu }\right)}^{3}\sum_{i=1}^{\infty }e^{-\mu_{i}^{2}\frac{\nu }{R^{2}}t}\left(\frac{\mu_{i}^{2}-6}{16\mu_{i}^{6}}-\frac{3\mu_{i}^{2}-18}{12\mu_{i}^{6}}\right)=\frac{t^{3}}{6}\sum_{i=1}^{\infty }e^{-\mu_{i}^{2}\frac{\nu }{R^{2}}t}+t^{2}\frac{R^{2}}{\nu }\sum_{i=1}^{\infty }\frac{e^{-\mu_{i}^{2}\frac{\nu }{R^{2}}t}}{\mu_{i}^{2}}+t{\left(\frac{R^{2}}{\nu }\right)}^{2}\sum_{i=1}^{\infty }e^{-\mu_{i}^{2}\frac{\nu }{R^{2}}t}(\frac{1}{\mu_{i}^{4}}+\frac{\mu_{i}^{2}-5}{6\mu_{i}^{4}}-\;\frac{3\mu_{i}^{2}-18}{6\mu_{i}^{4}})+4{\left(\frac{R^{2}}{\nu }\right)}^{3}\sum_{i=1}^{\infty }e^{-\mu_{i}^{2}\frac{\nu }{R^{2}}t}\left(\frac{3\mu_{i}^{2}-18}{48\mu_{i}^{6}}-\frac{12\mu_{i}^{2}-72}{48\mu_{i}^{6}}\right)=\frac{t^{3}}{6}\sum_{i=1}^{\infty }e^{-\mu_{i}^{2}\frac{\nu }{R^{2}}t}+t^{2}\frac{R^{2}}{\nu }\sum_{i=1}^{\infty }\frac{e^{-\mu_{i}^{2}\frac{\nu }{R^{2}}t}}{\mu_{i}^{2}}+\;t{\left(\frac{R^{2}}{\nu }\right)}^{2}\sum_{i=1}^{\infty }e^{-\mu_{i}^{2}\frac{\nu }{R^{2}}t}\left(\frac{1}{\mu_{i}^{4}}+\frac{-2\mu_{i}^{2}+13}{6\mu_{i}^{4}}\right)+{\left(\frac{R^{2}}{\nu }\right)}^{3}\sum_{i=1}^{\infty }e^{-\mu_{i}^{2}\frac{\nu }{R^{2}}t}\left(\frac{-9\mu_{i}^{2}+54}{12\mu_{i}^{6}}\right).$$

(A21) $$D=\frac{t^{4}}{24}\sum_{i=1}^{\infty }e^{-\mu_{i}^{2}\frac{\nu }{R^{2}}t}+\frac{t^{3}}{6}\frac{R^{2}}{\nu }\sum_{i=1}^{\infty }e^{-\mu_{i}^{2}\frac{\nu }{R^{2}}t}\left[\frac{2}{\mu_{i}^{2}}-\sum_{\begin{array}{c} j=1\\ j\neq i \end{array}}^{\infty }\frac{1}{\left(\mu_{i}^{2}-\mu_{j}^{2}\right)}\right]+\frac{t^{2}}{2}{\left(\frac{R^{2}}{\nu }\right)}^{2}\sum_{i=1}^{\infty }e^{-\mu_{i}^{2}\frac{\nu }{R^{2}}t}(-\sum_{\begin{array}{c} j=1\\ j\neq i \end{array}}^{\infty }\frac{1}{{\left(\mu_{i}^{2}-\mu_{j}^{2}\right)}^{2}}-\;\frac{2}{\mu_{i}^{2}}\sum_{\begin{array}{c} j=1\\ j\neq i \end{array}}^{\infty }\frac{1}{\left(\mu_{i}^{2}-\mu_{j}^{2}\right)}+\frac{19}{6\mu_{i}^{4}}-\frac{\mu_{i}^{2}}{3\mu_{i}^{4}})+t{\left(\frac{R^{2}}{\nu }\right)}^{3}\sum_{i=1}^{\infty }e^{-\mu_{i}^{2}\frac{\nu }{R^{2}}t}(-\sum_{\begin{array}{c} j=1\\ j\neq i \end{array}}^{\infty }\frac{1}{{\left(\mu_{i}^{2}-\mu_{j}^{2}\right)}^{3}}-\frac{2}{\mu_{i}^{2}}\sum_{\begin{array}{c} j=1\\ j\neq i \end{array}}^{\infty }\frac{1}{{\left(\mu_{i}^{2}-\mu_{j}^{2}\right)}^{2}}-\frac{19}{6\mu_{i}^{4}}\sum_{\begin{array}{c} j=1\\ j\neq i \end{array}}^{\infty }\frac{1}{\left(\mu_{i}^{2}-\mu_{j}^{2}\right)}+\;\frac{\mu_{i}^{2}}{3\mu_{i}^{4}}\sum_{\begin{array}{c} j=1\\ j\neq i \end{array}}^{\infty }\frac{1}{\left(\mu_{i}^{2}-\mu_{j}^{2}\right)}+\frac{9}{2\mu_{i}^{6}}-\frac{3\mu_{i}^{2}}{4\mu_{i}^{6}})+{\left(\frac{R^{2}}{\nu }\right)}^{4}\sum_{i=1}^{\infty }e^{-\mu_{i}^{2}\frac{\nu }{R^{2}}t}(-\sum_{\begin{array}{c} j=1\\ j\neq i \end{array}}^{\infty }\frac{1}{{\left(\mu_{i}^{2}-\mu_{j}^{2}\right)}^{4}}+\sum_{\begin{array}{c} j=1\\ j\neq i \end{array}}^{\infty }\frac{1}{{\left(\mu_{j}^{2}-\mu_{i}^{2}\right)}^{4}}-\frac{2}{\mu_{i}^{2}}\sum_{\begin{array}{c} j=1\\ j\neq i \end{array}}^{\infty }\frac{1}{{\left(\mu_{i}^{2}-\mu_{j}^{2}\right)}^{3}}+\;2\sum_{\begin{array}{c} j=1\\ j\neq i \end{array}}^{\infty }\frac{1}{\mu_{j}^{2}{\left(\mu_{j}^{2}-\mu_{i}^{2}\right)}^{3}}-\frac{19}{6\mu_{i}^{4}}\sum_{\begin{array}{c} j=1\\ j\neq i \end{array}}^{\infty }\frac{1}{{\left(\mu_{i}^{2}-\mu_{j}^{2}\right)}^{2}}+\frac{19}{6}\sum_{\begin{array}{c} j=1\\ j\neq i \end{array}}^{\infty }\frac{1}{\mu_{j}^{4}{\left(\mu_{i}^{2}-\mu_{j}^{2}\right)}^{2}}+\frac{\mu_{i}^{2}}{3\mu_{i}^{4}}\sum_{\begin{array}{c} j=1\\ j\neq i \end{array}}^{\infty }\frac{1}{{\left(\mu_{i}^{2}-\mu_{j}^{2}\right)}^{2}}-\frac{\mu_{i}^{2}}{3\mu_{i}^{2}}\sum_{\begin{array}{c} j=1\\ j\neq i \end{array}}^{\infty }\frac{1}{\mu_{j}^{2}{\left(\mu_{j}^{2}-\mu_{i}^{2}\right)}^{2}}+\;\frac{9}{2}\sum_{\begin{array}{c} j=1\\ j\neq i \end{array}}^{\infty }\frac{1}{\mu_{j}^{6}\left(\mu_{j}^{2}-\mu_{i}^{2}\right)}-\frac{9}{2\mu_{i}^{6}}\sum_{\begin{array}{c} j=1\\ j\neq i \end{array}}^{\infty }\frac{1}{\left(\mu_{i}^{2}-\mu_{j}^{2}\right)}+\frac{3\mu_{i}^{2}}{4\mu_{i}^{6}}\sum_{\begin{array}{c} j=1\\ j\neq i \end{array}}^{\infty }\frac{1}{\left(\mu_{i}^{2}-\mu_{j}^{2}\right)}-\frac{3\mu_{i}^{2}}{4\mu_{i}^{2}}\sum_{\begin{array}{c} j=1\\ j\neq i \end{array}}^{\infty }\frac{1}{\mu_{j}^{4}\left(\mu_{j}^{2}-\mu_{i}^{2}\right)}).$$

As of today, the authors of this work did not find such a universal formula; however, this does not mean that it does not exist.
